# Spirit of the Foreign Periodicals, &c.

**Published:** 1836-01-01

**Authors:** 


### Spirit of the Foreign Periodicals, &c.

### On Monstrosities in General.

The meaning of the words, monstrosity and monster, is not easily defined. We cannot draw a satisfactory line of demarcation between the irregularities of animal formation so designated, and those minor ones, which are usually called anomalies of, or deviations from, normal structure.

In ordinary language, the acceptation of the word, monster, has reference almost solely to the distortion of the external shape or configuration of the being; and scientific men, while they acknowledge its imperfection, or rather erroneousness, have been obliged to retain it for want of one of more precise import. It is however to be well remembered, that the mere study of the outward and visible features of monsters can be of little use; that the bare enumeration and arrangement of them from such data can furnish but a vague alphabet of terms, and cannot throw any light on the obscure but very interesting subject of their etiology. It has been only within the last thirty years that the study of monstrosities has been pursued as a branch of science, and that it has led to any interesting results. There is no want indeed of marvellous cases in the folios of former days; but there is a want of a guiding principle to enable the reader to draw any useful conclusions from them.—Hence it has happened that, although the works of anatomists of every age have teemed with descriptions of monsters of every form and size, the field of enquiry was nothing but a "tabula rasa," until enlightened by the discoveries of such men as Meckel, St. Hilaire, and Serres.

It is but fair however to state, that to Littre, who lived at the commencement of last century, perhaps the great merit is due, of having first announced a rational explanation of some monstrosities. The principle on which this explanation is founded, and which has been so beautifully developed and amplified by the distinguished anatomists of France and Germany in the present century, is the simple one of regarding certain deviations of structure as the results, not of any unmeaning perversion, or chance confusion of formation, but of a mere arrest of development, during the successive changes which the organs invariably undergo in the fœtal state. Numerous examples illustrative of this most ingenious idea will be enumerated in the course of this article. To other monstrosities indeed the law of arrested development cannot be made to apply; but even with respect to these, the researches of Geoffroy St. Hilaire, and other philosophers, have accumulated some very striking facts, which may yet lead to a satisfactory solution of the problem of their formation.

If we take a general view of the various deviations of normal structure which organised bodies may exhibit, they may be all conveniently grouped either as irregularities of formation, or as irregularities of texture. The former occur only during intra-uterine life, and before the being is perfectly developed; while the latter occur most usually after birth. True it is that we speak of postgenital irregularities of formation; but they are essentially different from those now under consideration, and are, properly speaking, merely the results of a previous change of texture, under the operation of which the organs may lose their normal shape.

The period of intra-uterine life at which monstrosities originate must differ much, according to the nature and extent of these deviations. Because a fœtus is born monstrous, it does not necessarily follow, that the being had never a normal form. It is quite probable that the form may have been strictly regular during the early stages of evolution, and may have become modified at a later period in consequence of some error of the ulterior development.

Now this error of development may have taken place in several different ways: sometimes the formative power (to use a German expression) has less energy than is usual; and then the development of the organ or organs is checked; they are imperfect, or altogether deficient. At other times this formative power has been unusually energetic; and then the organs will exhibit an excess in size, or a redundancy in number. Or, lastly, it appears that the formative power may undergo a simple perversion, without being accompanied with either an excess, or a defect of development; and hence modifications in the direction and position of organs, more or less important, may be induced: as examples of such a perversion, we may mention a general transposition of the viscera, certain irregularities in the origin of the arterial trunks, &c.

Under one or other of these three great sections, may be arranged every variety of monstrous growth.

However numerous and varied monstrosities may be, it is very apparent that nature obeys certain laws and limits, even in the case of these very exceptions from her ordinary works. We may be quite assured, that there is no such thing as fortuitous, or haphazard contingencies, even in *her* most wayward irregularities. No one has ever heard of such an extravagant malposition as that of the lungs being developed in the cranium, or of the brain being formed in the pelvis; and again, however abnormal the deviations of a monster may be, yet never is there such an utter confusion of organs, that a mucous and a vascular tube, such as the intestines and the aorta, are continuous with each other. All this however might happen, if the forming energy was not subjected to certain laws during the apparent disorder of its action.

So far from there being any chaotic confusion of parts, even in the most abnormal of monstrous growths, it is a most curious and highly instructive fact, that many of these very irregularities occurring in the fœtuses of man and of the higher animals, are in truth the natural formations in some of the lower tribes of animals. Thus, for example, the human brain, if arrested at a certain period of its evolution, may appear more or less exactly analogous to the normal condition of the organ in fishes or in reptiles; and many of the congenital malformations of the heart are almost exact types of this organ in these tribes of animals. The converse, however, of this proposition is never true: we mean to say, that never does the brain, or any other organ, in these inferior animals, attain a degree of development at all equal to the natural development of the part, in the animals which are above them in the scale of existence.

Whenever the deformity of a monster is considerable, we almost invariably find that there is more than one part or organ of its body abnormal in structure and configuration. The deviations may all belong to the same class or section: for example, all of them may consist in either a deficiency or an excess of the natural development. To such monstrosities Meckel has applied the epithet of compound or composite; whereas those which exhibit errors of formation, of different sorts, he terms "complicated."

The complicated monstrosities are of more frequent occurrence than the compound. The cause of this greater frequency is doubtless attributable to the operation of that law, so well illustrated by M. St. Hilaire, in virtue of which the excessive nutrition of one organ induces a greater or less degree of atrophy of another, and vice versâ.

Numerous are the applications which may be made of this law of compensation (balancement) in the study of teratology. Thus it is not uncommon, that when one hand or foot is furnished with supernumerary fingers or toes, the other corresponding member has not its natural number. It is worthy of notice here that a redundancy of fingers and toes is not an unusual occurrence, whenever there is an incomplete development of some important organs of the body. Meckel has made this remark of cyclopic or monocular monsters.

When the encephalon is more or less deficient, there is very commonly an excessive development of the face.—When the lower extremities are imperfectly formed, the number of the vertebræ and of the ribs is often increased. When the heart and liver are deficient, as is frequently the case in acephalous monsters, the size of the kidneys is generally much exaggerated. This same law of compensation may be traced in the conformation of such monsters, as have some important part doubled: thus when there are two trunks, the head is often wanting; and on the other hand, in bicephalous monsters, the spine is often bifid. In such cases, other parts and organs are usually imperfectly formed: thus the abdominal integuments may be utterly awanting; the intestinal canal may be incomplete; the urethra be imperforate; and the rectum and bladder may terminate in a common cloaca.

Another application suggested by Meckel of the law of compensation, but certainly one of doubtful propriety, is that in the different members of the same family a monstrosity of excess in one may be, as it were, compensated for by a monstrosity of defect in another. Thus a case is reported by Morand of a girl who had a supernumerary digit on each hand and foot. One of the hands of this girl's sister was fingerless.

With respect to the comparative frequency of monstrosities "par exces, ou par defaut," in different parts of the body, we may state it as a general rule, that the external parts are much more frequently increased in point of number than the internal parts. It is rarely that we find more than one heart, one intestinal canal, two lungs, or that a double genital or urinary apparatus exists; whereas few monstrosities are more common than that of supernumerary extremities.

Meckel has announced, (shall we say established?) the general law, that the organs, or apparatus of organs, whose nerves are supplied from the cerebro-spinal axis, as the muscular system, the larynx, and lungs, are less frequently malformed, than those which derive their nerves from the great sympathetic, to wit, the digestive, the genital, the urinary, and especially the vascular system. This law, if established, is the more remarkable, as the cerebro-spinal system itself is much more liable to changes of conformation than the ganglions of the great sympathetic.

Some irregularities of formation are as frequent on one side of the body as on the other; for example, this is true of redundant, or deficient digits: others "affectent une singuliere predilection" for the left side. Meckel has made the remark that, when the vertebral artery arises directly from the aorta, it is almost always the left one. Monstrosities are of more frequent occurrence in the upper than in the lower half of the body: supernumerary fingers are more common than supernumerary toes.

Haller and Meckel have concluded from their researches, that monsters of the female sex are greatly more common than those of the male. Of 80 examples collected by the latter anatomist, three-fourths of the number occurred in female fœtuses. The cause of this difference is probably to be sought for in the curious fact that, during the earlier stages of the evolution of the being, there is but one sex, viz. the female, as is normally the case in the lower members of the animal scale. To say, therefore, that the greater number of monsters are of the female sex, is merely equivalent to affirm that, in most of them, the development of the genital organs has been arrested at an early period of fœtal life.

That certain monstrosities, such as the existence of supernumerary digits, are of hereditary communicability, appears to be very probable from a multitude of examples recorded by Meckel, Morand, and Osiander. It has been remarked that, double-headed or double-bodied monsters, have proceeded more frequently from women who have had twins before, than from those who never gave birth to more than one child at a time.

After these preliminary general observations, we shall now briefly review some of the most interesting and best ascertained facts, in reference to each section of the ternary division of monstrosities; and, first, with respect to monstrosities "par defaut."

The organs of the body which are most frequently found defective, are those whose normal development is known to be the most tardy. One of the organs most early evolved is the intestinal canal; being at first simply a continuation of the vesicula umbilicalis, it is gradually prolonged into two portions, an upper (the small intestine and stomach) and a lower (the large intestine.)

Now, in every monster hitherto examined, some traces of an intestinal tube have been found. The primordial portion of it is invariably present; whereas the other portions of it, which we know to be of subsequent growth, are not unfrequently defective, or even entirely absent. If the upper or lower prolongation has been arrested at an early period of its evolution, it will be found to be of very short extent, and to terminate in a cul-de-sac. The stomach being evolved subsequently to the small intestine, we can readily understand how the former viscus may be either altogether awanting, or so imperfect, that it looks only like a continued portion of the small gut. On the same principle, we may explain the more or less complete deficiency of the rectum, and several other congenital malformations, not unfrequently observed in monstrous fœtuses.

Whether the urinary bladder is, or is not, the continuation of the allantoid membrane, it is known that this viscus, and also the kidneys are developed at a very early period of fœtal life. Hence we may, à priori, conclude that the urinary organs will be seldom, or almost never, utterly awanting. Observation shews the correctness of this conclusion; for, in the most imperfect and deformed monsters, some traces of a urinary apparatus are almost invariably found.

The vascular and nervous systems, also, are very early developed. Their primary lineaments are found in the apparently homogeneous mass of which the very young embryo consists; and their perfect development being slow, gradual, and not completed even at the end of the ninth month, the irregularities of their formation are among the most common of all abnormal deviations of animal structure. Both of these great systems may be considered as formed by the successive re-union of several secondary systems, each of which has, in some degree, an independent develepement. Thus, in the case of the nervous system, it has been demonstrated that the nerves, instead of issuing from the cerebro-spinal axis, are actually formed before it. Now, in accordance with the law which we have been illustrating, we find that irregularities of the great nervous centres are of more frequent occurrence than irregularities of the nerves themselves. The spinal marrow being developed anteriorly to the encephalon, the former is more rarely deformed than the latter; and, with respect to the encephalon by itself, the very parts of it which are of latest formation are most subject to congenital alterations of size and form.

It was long a prevalent notion, that the heart is one of the parts of the body which is most early developed. This is quite a mistake. Many of the blood-vessels are formed before there is any vestige of a heart apparent; and when it does appear, its structure is very different from what it is at the ulterior periods of fœtal existence. At first it is only a simple tube or canal continuous with the aorta or dorsal vessel. Gradually it swells or bulges out into a rounded sac, consisting of but one cavity. The division of this cavity into separate chambers is an act of subsequent development. Now it is in those very parts of the organ which are of latest formation, that monstrosities or irregularities of structure are most frequently observed.

The persistence of the patency of the foramen ovale, after birth, is the most common of these irregularities. More rare is the imperfection or deficiency of the ventricular septum—a state of things, however, which is the normal condition of the human heart, at an early period of fœtal life.

At a still earlier stage, there is no obvious distinction between the auricles and the ventricles; and hence this disposition of parts is still more rare in monsters than the preceding one. The most rare of all cardiac malformations is the existence of a simple tube or vessel in the place of the heart, such as we observe in the heart of all fœtuses when the organ is first appreciable, and such, also, as is the normal condition of the heart in some of the inferior tribes of animals. Thus, these different malformations afford a most convincing illustration of the operation of arrested development, as the cause of their production.

If our limits permitted us, we might extend our observations to the osseous and other systems of the body, and we should find innumerable examples confirmatory of the law, that those parts which are of latest evolution are the most frequently malformed or monstrous; and vice versâ.

If, from the consideration of individual organs, we turn our attention to the external "ensemble" of the body, and to the great regions or segments of which it is composed, we shall discover that those regions which are most tardily developed are more frequently incomplete or entirely absent than the others, which have preceded them in evolution. In the very early period of gestation, the embryo appears to consist altogether of abdomen. Now no monster has ever been found, in which this section of the body, more or less perfect, has been deficient; the head, thorax, and extremities are occasionally entirely absent, but invariably there is something of an abdomen to be discovered, just as we observe to be the actual and normal state in the commencement of embryotic existence. The converse of this proposition, viz. that the head and thorax may be formed without any vestiges of an abdomen, has never been verified. It is opposed alike by physiological reason, and by the results of anatomical research.

The genital organs being late of formation, imperfections of these are among the most common of all monstrosities. The eye precedes the ear in development, and observation assures us that it is less subject to congenital defects than the latter organ.

The recent enquiries in human and comparative embryology have clearly shewn us that most organs, in their formation, are much more independent of each other than was generally believed before; and that, consequently, the arrested developement of one organ does not necessarily induce a similar condition in any other. For example, we now know that the nerves are formed independently of the encephalon and spinal marrow. Numerous cases of anencephalie and amyelie (derived from α, priv. and μυελος, medulla) establish this position.

The nerves seem to be formed primarily in the substance of the different organs themselves, and are only subsequently joined to the great nervous centres, with which they are to be so intimately connected in after-life. The developement, therefore, of the nerves is much more dependent on the presence of the organs to which they belong, than on the presence of the brain and spinal cord. A double-headed monster, examined by M. Serres, afforded an apt illustration of this law: although there were two brains, there were not more than two pneumogastric nerves—and why? because, there being but one body, not more than one pulmonary and digestive apparatus was present. In the opposite kind of monstrosities, viz. where there are two bodies, and only one head, M. Serres has always found a quadruple pneumogastric nerve—two to each body.

This ingenious physiologist has contributed much to the science of teratology. The hypothesis which he has advanced to explain the mysterious subject of monstrosities, although supported by many plausible arguments, has not met with very general adoption. He supposes that all deviations in structure and form of any organ is necessarily preceded, and indeed caused, by some irregularity or deficiency of the bloodvessels which are destined for its supply. According to this doctrine, the bloodvessels of every part are first evolved, and the rest of the structure subsequently developed. If, therefore, these vessels, which convey the materials of growth and nutrition to the organ, be abnormal in number or distribution, it is believed that the organization of the part is necessarily deranged and altered. The more early the vessels of any part of an organ are formed, the more precocious, he says, will be the development of that part. Again, if the vessels be from any cause interrupted, or altogether deficient, the organ or member, which they are destined to nourish, will be incomplete or absent. M. St. Hilaire has adopted this hypothesis, and has laboured hard to establish it. But it must be acknowledged that it is founded on an assumption, viz. that the bloodvessels of a part must be always formed before any other structure; and the objection of Beclard, that it is impossible to determine which is really the cause, and which is the effect of the coincident phenomena, cannot be easily refuted. Indeed, some facts seem to be more favourable to the very reverse of Serres' hypothesis; thus, for example, in the organization of false membranes or morbid deposits, the bloodvessels appear to be developed in the very substance of these, at first as isolated red points, which gradually lengthen out into tubes, these tubes ultimately anastomosing with each other and with the adjacent vessels; cases, also, of anencephalie are on record, where not only the internal carotid, but even traces of its cerebral branches were nevertheless discovered.

So much for the law of "sanguiferous developement," which M. Serres has so ably endeavoured to establish.

There is another hypothesis respecting monstrosities which this scientific anatomist has advanced, to explain most of those "vitia confirmationis" which consist in fissures or divisions of organs which, in the mature being, are united and integral. Almost all these fissures are situated along the median line of the body.

This uniformity of position is ingeniously accounted for by the fact, that at an early stage of embryotic existence, the greater number of the organs are composed, in fact, of two portions, the interval between these portions gradually contracting until it is obliterated during the progress of development. These changes M. Serres refers to the more general law of concentric developement, in virtue of which all parts of the body are formed from the periphery to the centre, and not from the centre to the periphery. If, therefore, the evolution be arrested, before the obliteration of the fissures or divisions of the organs has taken place, it must follow that they will appear as bilobed or bifid at the period of birth. Many of the congenital deformities affecting the cranium, the lips, the spine, sternum, abdominal parietes, pelvis, male genital organs, &c. may be adduced in favour of this hypothesis of M. Serres. All these monstrosities, therefore, are to be regarded as the results of an arrested development, at some period or another of fœtal life, and not, as was long supposed, of any disease—far less of a fortuitous growth. In the same manner may be explained most of the opposite class of monstrosities—those which consist either in the occlusion of passages normally open, the "atresiæ" (from α, priv. τραω, perforo), or in the reunion or concretion of parts normally divided, the "symphyses" of authors. To this class belong the imperforate condition of the rectum and urethra, the termination of these canals in a common duct, the persistence of the membrana pupillaris, &c.

Another great class of monstrosities, viz. those which consist in the inordinate size or multiplication of parts, may, at least in numerous instances, be traced to the same great law of arrested developement, which it has been the chief object of this article to illustrate. At present, we cannot do more than merely allude to them. Deviations, also, from the normal direction, and from the normal colour of organs, have almost all their prototypes, in the conditions which are natural to the fœtus at some period or another of its early life.

The important discovery that these varying conditions, or states of organization, which the fœtus exhibits at different periods of its existence, and, consequently, that most congenital monstrosities also, are analogous to, and closely represent, the normal structure of animals lower than the mammalia in the scale, adds a great interest to teratological pursuits. That the human being, during its fœtal life, passes successively through several stages of organization, [which are the permanent conditions of animals below it in the scale], from a very simple to the more complicated formation when it arrives at mature developement, is established by a multitude of facts. A few must suffice us at present. The imperfection or absence of the extremities finds a parallel among the cetacea, and among many reptiles and fishes. The various changes of structure which the heart and large bloodvessels exhibit, and to which we have alluded at a former part of this article, afford still more striking illustrations. The heart, "allongé en vaisseau," of the young fœtus, and of some acephalous monsters, may be compared to the dorsal vessel of insects; when it forms a unilocular muscular sac, we are reminded of the heart in the crustacea; where there are two cavities—of the heart in fishes and many mollusca; where there are three (two auricles and one ventricle)—of the heart in the batrachia, and so forth.

Numerous examples may be drawn, also, from the alimentary, urinary, and genital organs. The imperforate state of the rectum and urethra, the termination of these passages in a common cloaca, the imperfect developement of the stomach, the absence of the gall-bladder, the lobular and united state of the kidneys, the large size of the thymus and supra-renal glands, the permanence of the testicles in the abdomen, the bicorned state of the uterus, &c. all these are represented, it is well known, by the normal conditions of the viscera in question, in different tribes of animals. If our space permitted, we should draw other proofs from the irregularities of developement which are observed in the osseous system. We must plead the same apology for the abrupt termination of these remarks, leaving unconsidered the second great department of monstrous growths, namely, those which consist in a preternaturally increased size or number of greater or less portions of the body. In our next Number we shall endeavour to complete the subject.

---

### Case of Monstrosity.

#### Lateral Transposition of the Viscera—Great Cardiac Malformation, without Cyanosis.

The infant, the subject of the present case, was born with a double harelip, complicated with a separation of the intermaxillary from the left maxillary bone, for the extent of an inch. The mouth and left nostril being thrown into one cavity, there was a large gap at the fissure, through which the tongue and pharynx could be distinctly seen. The os incisivum closely approximated to the right maxilla was covered with a triangular flap of the lip, which adhered firmly to it. The left portion of the lip adhered so strongly to the left maxillary bone, and the separation of the osseous parts was so considerable, and extended so far backwards, along almost the whole length of the palate, that little could be done to remedy the deformity by operation. The velum palati also was bifid.

This infant presented no other outward irregularity of formation. It was large, vigorous, and emitted a tolerably distinct cry. The colour of the surface was quite natural. For some days, it seemed to thrive sufficiently well; it slept quietly, and the breathing was not disturbed, except when it attempted to swallow.

On the eighth day after birth, the operation for the harelip was performed. For some hours afterwards, the little patient did not appear much distressed; but, towards evening, the breathing became slow and very laborious, and death took place on the following morning, but without any appearance of cyanosis having ever been manifested.

*Dissection.* The heart was found to be situated in the right side of the thorax, its apex pointing to the right fifth intercostal space: the arch of the aorta crossed from the right to the left side, and the vessel descended along the right side of the dorsal vertebræ. The two auricles seemed to form one large distended pouch, situated behind the ventricles; the appendices were elongated to a very unusual extent. There were two superior venæ cavæ, terminating, the one in the right, and the other in the left side, of the large auricular pouch, which received also the inferior cava and the pulmonary veins. The ventricles were most disproportionate in size —the left one was large, and the right one appeared as a mere appendage to it. On laying open the cavities, the septum was found to be imperfect towards the base. To the right of the aperture of the aorta, there was another aperture, which admitted the point of the finger, and which established a communication between the two ventricles. The right ventricle, from which the pulmonary artery arose, had no communication with the common auricular pouch or with the venæ cavæ, and, therefore, it must have received the blood, to be afterwards discharged into the pulmonary artery, only through the orifice which existed between it and the other ventricle. In short, it seemed to be rather a mere appendage to the left ventricle, than one of the regular cavities of the heart. The ductus arteriosus and the branches of the pulmonary artery did not exhibit any unusual appearance. On examining the internal surface of the large common auricle, there was observed a median longitudinal fold; the rudiment, no doubt, of the inter-auricular septum. By this fold alone, we were enabled to distinguish the right auricle from the left one. The orifice of one superior cava was found to the right of the fold, and that of the other to the left. The inferior cava also, and the pulmonary veins, terminated on the left side of the fold. The large common auricle had only one auriculo-ventricular orifice, guarded by an imperfect tricuspid valve, and leading into the aortic or left ventricle. The heart, therefore, in this child may be viewed as consisting of but two cavities—an auricle and a ventricle, such as is the normal condition of the organ in the batrachian tribe of animals. Most of the abdominal viscera were more or less malformed or displaced. The gall-bladder was situated to the left of the umbilical vein; the inferior cava was on the left side of the vertebral column; the vena portæ entered the liver at the left extremity of the transverse fissure: the greater portion of the small intestines was found in the left flank; the cæcum, however, was in its natural situation, but the colon, after ascending a little way, was soon reflected upon itself, so that the sigmoid flexure was on the right side of the abdomen. One of the most singular characters of the present case was the utter absence of the spleen, and, on examining the cæliac artery, it was found that it gave off two branches only, the hepatic, and the superior mesenteric.

The preceding case is one of great interest, as illustrating what extent of malformation of the heart is compatible with a limited duration of life. The entire absence of any cyanotic discoloration of the skin, and the comparative healthfulness of most of the functions under such circumstances, deserve particular notice. The respiration was never much distressed, except when the infant attempted to drink; but this, be it remembered, is very commonly the case, whenever the upper lip is cleft to a considerable extent, and the maxillary bones are separated from each other.

The hypothesis very generally held, that the symptoms of cyanosis are attributable solely to the commixture of the venous and arterial blood in an imperfectly closed heart, is shewn to be greatly erroneous. When the foramen ovale is merely patent, it is quite possible, as Meckel and others believe, that the two currents of blood may remain nearly altogether distinct; but such could not have been the case in the infant whose history we have reported, as the blood from the two cavæ and from the pulmonary veins was mixed together in the common auricle.

The curious tendency to a lateral transposition of most of the organs in some monsters has not been satisfactorily accounted for. Arrest of development cannot surely be the real cause; for, although during fœtal life several organs, such as the heart, spleen, &c., are placed nearer the median line than after birth, no one has ever supposed that there is any positive change from one side of the body to the other, during the various phases which the young being undergoes.—*Dictionnaire de Médecine et Archives Générales.*

---

Obliteration of the Aorta, common Iliacs, &c.

The patient, a woman, 51 years of age, was admitted last March into the La Charité Hospital, under the care of M. Louis. In 1812, she had suffered from a severe attack of rheumatism in the upper and lower limbs, which had left great weakness and muscular inactivity for upwards of a year and a half. In 1831, a numbness of the right leg came on without any appreciable cause: four months afterwards it affected the left limb: this symptom was always much aggravated by walking and other exercise. During the following year she began to experience dyspnœa and palpitations of the heart. All these troublesome symptoms had gradually increased, so that for the last year and a half she had scarcely ever left the house, and she had been obliged to sleep in an almost sitting attitude.

She was suffering at the time of admission from dyspnœa, and strong palpitation of the heart: there was a general yellowness of the surface; the lips were purple; the superficial veins, especially of the neck, were remarkably distended; there was no œdema of the lower limbs; the general health was so much debilitated, that gentle exercise of any kind soon distressed her. Mild aperients and diuretics were ordered.

The action of the heart gradually became more tumultuous and irregular, and the pulse was extremely quickened; the breathing was more oppressed, and a troublesome cough, accompanied with copious expectoration, partly bloody, came on. These symptoms became daily aggravated, and the patient was at length forced to be continually sitting upright, the dyspnœa and palpitations of the heart being so dreadfully violent: at each pulsation of the heart, the parietes of the chest were forcibly elevated, and on applying the hand over the cardiac region, a distinct "fremissement" could be readily felt. She died on the 10th day after her admission into the hospital.

*Dissection.* The pericardium contained about two ounces of a reddish coloured serum. The heart was found considerably enlarged in volume and hypertrophied: the left auriculo-ventricular orifice was contracted, so that the fore-finger could not be passed through it; its border was hard and thickened, and the mitral valve also was similarly diseased; the aortic semilunar valves were rigid and cartilaginous.

The aorta itself, from its origin unto the giving off of the renal arteries, appeared quite normal; below this point to its bifurcation it was contracted into a stiff unyielding solid cord; the iliac arteries, and their two great branches, were similarly affected, feeling under the finger as ligamentous cords.

The aorta having been slit open, it was found that its canal was quite pervious and free, until the point where the renal arteries were given off. At this point it was filled with a lamellated coagulum, which adhered to every part of its circumference except for about the breadth of a third of an inch posteriorly. The coagulum extended downwards to the point of bifurcation, and was thence continued along both common, internal, and external iliac arteries, adhering very strongly to the internal surface of the vessels at some points, and but loosely at others. How far the coagula extended along the internal iliacs or hypogastrics could not be satisfactorily determined, as the vessels had previously been divided in removing the pelvic viscera: in the right hypogastric, the coagulum was traced for upwards of an inch. In the left external iliacs the coagulum was traced downwards, becoming gradually smaller and more tapering unto an inch below Poupart's ligament, where it ceased; on the right side, it traversed the whole extent of the femoral, sending off a division into the profunda femoris to the popliteal artery, and was thence continued along the posterior tibial, one half way down the leg, where it terminated in a few loose lamellæ.

The anterior tibial artery remained free. In the left popliteal there were several thin smooth lamellæ adhering to the internal surface of the vessel. It is not to be inferred that wherever the coagulum was found, it was a solid impervious substance, and that the canal of the artery was altogether plugged up. In the right and left common iliacs it lined the internal surface, forming a hollow cylinder, which terminated in a cul de sac superiorly at the bifurcation of the aorta, and inferiorly at the division of the vessel into the external and internal iliacs. Lower down in the external iliacs, the coagulum again became tubular and pervious, as far as Poupart's ligament on the left side, and on the right side along the whole extent of the femoral and popliteal vessels: in the posterior tibial, the coagulum was nearly solid throughout.

With respect to the abdominal branches of the aorta, the trunk of the cæliac was quite free: the splenic artery was partially obstructed by lamellæ of coagulum: so also was the superior mesenteric, after giving off one branch; the lumbar arteries were empty, but their orifices were plugged up; and the inferior mesenteric was obstructed "par un coagulum peu volumineux; le stylet ne peut traverser son orifice."

The canal of the middle sacral artery was pervious; but its orifice was plugged up. The right brachial also was partially obstructed by a coagulum, which adhered to its internal surface.

The internal coat of those arterial tubes, which were obstructed exhibited signs of a morbid change to a greater or less degree, according to their proximity to the bifurcation of the aorta, where these signs were most strongly developed: it was thickened, rugous, opaque, "peu consistante," and therefore more easily lacerable than in a state of health. These changes were most conspicuous wherever the coagulum was solid, impervious, and adherent most strongly to the inner surface of the vessel.

M. Barth, the reporter of the case, attributes the alteration of the arterial tissue to an inflammatory action, the consequence of which had been the deposition of a thin layer of coagulable lymph, either secreted from the vasa vasorum, or derived from the contained blood: the blood in consequence being partially impeded, began to deposit fibrinous layers, which gradually accumulated until the cavity of the vessel was more or less completely obstructed.

The coagulum once formed, the arterial parietes contract upon themselves, in accordance with the law of the vascular system, that whenever there is any obstacle to the free circulation of the blood, the vessels gradually close, until they become impervious ligamentous cords.

M. Barth is of opinion, that the disease of the bloodvessels commenced four years before her decease, at the period when she first experienced the numbness in the right leg and thigh. The formation of the coagulum being slow, the progress of the symptoms was consequently very gradual.

At what point the morbid change commenced, it is not possible to determine; but judging as well as we may from the post-mortem appearances, it seems probable, that the lower extremity of the aorta (between the giving off of the inferior mesenteric and the point of bifurcation) and the common iliacs were first diseased.

M. Barth proceeds to offer some considerations respecting the mode in which the circulation was most probably carried on, to compensate for the obstruction of the great arterial trunk in the lower half of the body. It is unnecessary to follow him through this disquisition, as the data are rather unsatisfactory. We may however state that the mammaries, the epigastric, intercostals, gluteals, &c. did not *seem* to be much enlarged in volume. It is to be remembered, however, that these vessels were quite empty, and had not been injected with wax, as in the well-known experiments of Sir A. Cooper on dogs, in which the aorta had been tied.

In the annual report of the proceedings of the anatomical society of Paris for 1834, allusion is made to some interesting examples of partial and complete obliteration of the large arterial and venous trunks.

In one case, the right common and external iliac, and the crural arteries were found completely obstructed by sanguineous clots; the aorta also was partially obliterated. These vessels had evidently suffered from inflammation; their internal coats being highly injected and thickened, and traces of purulent matter, deposited either in the centre of the coagula, or on their surfaces, being found at different points.

In other cases, the obstructed arteries exhibited no appearances "d'une inflammation actuelle." The brachiocephalic trunk was once found almost entirely plugged up by a fibrinous clot.

Allusion is made to another example of the obliteration of the iliacs and of the lower end of the aorta.

An interesting case of arteritis, connected with gangrena senilis of the foot, occurred in an old woman who died recently at the Hotel Dieu.

Violent pains had been felt along the whole length of the affected extremity: these pains were always aggravated by the application of any stimulants. On dissection, the anterior tibial artery and its branches were found converted into fibrous cords: the crural and iliac arteries also were nearly quite obliterated: the inferior part of the aorta was partially so by a coagulum, in the centre of which was some purulent matter. The parietes of the affected vessels presented evident marks of having suffered from inflammatory action.

Although the following case of obliteration of the pulmonary artery is one not of vascular disease, but of congenital malformation, it will be read with interest. The infant was taken to a hospital, in consequence of "un endurcissement du tissu cellulaire:" the articulations were stiff; the pulse of the affected members was imperceptible; nothing abnormal in the pulsations of the heart could be discovered. The infant died on the 12th day after birth.

On dissection, the greater part of both lungs was found in a hepatised state. The heart was much larger than usual. The two ventricles communicated freely with each other at their upper part; and from about the centre of this part arose the aorta considerably enlarged. The pulmonary artery was not half the size of the aorta, and its tube formed a cul de sac towards the right ventricle, on whose inner surface no trace of any opening could be discovered. At a distance of eight lines or so from this ventricle, the pulmonary artery gave off some branches, and at this part there was a free communication between it and the aorta, by means of a ductus arteriosus.—*Archives Générales.*

---

### Obliteration of the Vena Cava.

In our respected Edinburgh cotemporary for last April, a most interesting example of this very rare lesion is related. It was discovered in the body of a female, who had entered the hospital with symptoms of hydrothorax and general anasarca. She died soon after admission.

On dissection, a large quantity of serum was found in the right pleural cavity: the kidneys were diseased. The tube of the superior vena cava was obliterated, and the vessel converted into a hard cartilaginous cord, from its junction with the right auricle, for the extent of two inches. Around this cord, especially at the lower part, there were numerous calcareous concretions. On examining the inner surface of the auricle, the opening of the vena cava was indicated only by a slight depression.

Unfortunately the dissection of the body had been already rudely performed, when this very curious lesion of the heart and vena cava was detected, so that it was not easy to ascertain exactly the state of the other contiguous parts of the vascular system.

The vena azygos was greatly enlarged, but its termination in the cava superior was quite obstructed: all the intercostal and lumber veins also were much dilated; and the vena cava inferior, which must have returned all the venous blood of the body with the exception of what was contained in the coronary veins, was so large, that it admitted readily three fingers, at its termination in the right auricle.

Allusion is made by the narrator of the preceding case to a curious anomaly of the abdominal venous trunk which he once found. The common iliac veins, instead of uniting at the usual point to form the cava inferior, ascended separately to the level of the renal veins, where they joined together. The trunk thus formed passed through the aortic opening of the diaphragm in the place of the azygos vein, and terminated in the upper cava, before its entrance into the right auricle.

Dr. Deckart has related, in a thesis published at Berlin in 1823, an instance of the complete obliteration of the superior cava, the parietes of which vessel intimately adhered to, and were confounded with, an immense aortic aneurism.

Two similar cases are recorded, the one by Dr. Otto in his "Observations rares Concernant l'Anatomie," and the other by Dr. Reynaud, in the Journal Hebdomadaire for January, 1829.

In a case related to the Anatomical Society of Paris by M. Tessier, the iliac and crural veins were found strongly inflamed. At the entrance of the iliac into the vena cava, there was a false membrane, which completely obstructed the passage of the blood between the cava and the inflamed veins.

In another case of spontaneous phlebitis of the pelvic extremity, a large clot plugged up the canal of the common iliac vein, and had thus prevented the admixture of the purulent matter which existed lower down in the crural vessels with the blood of the vena cava.

In a patient who had died of ascites, the vena portæ was found quite obstructed by a sanguineous clot. (Was the dropsical disease the result of the obstruction? In part, probably.)—*Archives Générales.*

---

### On the Auscultatory Signs of Pregnancy.

The sounds perceived on applying the ear to the abdomen of an unimpregnated female are in the majority of cases, merely the rumbling and rolling noises of the gaseous and fluid contents of the bowels, and the pulsatory beats of the aorta, or of some other of the large blood-vessels. The former of these sounds is too characteristic to be mistaken for any other; and the latter is a simple, uniform beat, unaccompanied usually with any distinct blowing, or whizzing noise. This pulsatory sound is generally most distinct in the epigastric region on the left side. A similar sound is not unfrequently perceptible in the corresponding part of the right side, and proceeds probably from the pulsations of the axis cœliaca. In the left iliac region also, a pulsation, accompanied sometimes with an indistinct murmuring, or humming sound, is often heard. This proceeds from the left iliac artery, and the cause of the humming sound is probably to be sought for in the relative position of the large artery and vein here, the one being somewhat underneath, and being somewhat compressed by the other.

Dr. Hohl has examined by auscultation many women, during the period of the catamenial flow; but in no instance has he detected any peculiar sound.

In women recently delivered, and especially when the uterus has not yet completely contracted, and strong after-pains are present, Dr. H. has found that, at the part where the placentary bruit had been heard during pregnancy and labour, a feeble humming sound may generally be perceived. It is somewhat more distinct at each beat of the pulse at the wrist, and it succeeds, by a very short interval, the pulse of the heart. When the uterus is hard, and has contracted powerfully, and the after-pains have ceased, this humming sound is heard no longer.

Dr. H. has communicated the results of his auscultatory experience in certain cases of abdominal disease, as enlargement of the liver, in dropsy, and other lesions of the ovaries, ascites, tympanitis, &c., but as they do not establish any important practical conclusions, we shall not dwell upon them.

In the early months of pregnancy, the rumbling rolling noises from the contents of the bowels are heard as in the unimpregnated abdomen. After the sixth or seventh month, they become much more indistinct; and in the eighth and ninth months, they are perceptible only at the sides of the abdomen. This change is owing to the gradual displacement of the bowels, in consequence of the enlarging uterus rising and pushing them upwards and backwards.

In cases, therefore, of doubtful pregnancy, it may be worth while to attend to the circumstance now mentioned. It is indeed of minor importance, and can be admitted only as an auxiliary symptom; but the success of a physician often depends upon attention to some apparently trifling incident, which is neglected by others. We come now to the consideration of the characteristic or pathognomonic auscultatory signs of pregnancy.

It is admitted by all obstetrical auscultators, that two sounds, which are not audible in an unimpregnated female, are to be heard in the abdomen of a pregnant female, at least after a certain period of pregnancy. The one is a simple, uniform, and dull sound, accompanied usually with a blowing, whizzing, or murmuring noise, and its recurrences are synchronous with the beats of the woman's pulse. The other is much more rapid and sharper than the former; it is double, resembling the tic-tac of a watch, and its recurrences are quite independent of, and are very seldom or never synchronous with, the beats of the maternal pulse. These two sounds, therefore, are so dissimilar in all their characters, that they cannot possibly be mistaken the one for the other. As to the seats of these sounds, the prevailing opinion has hitherto been, that the first or blowing sound proceeds from the passage of the uterine blood into and through the placenta. For this reason it has been denominated the "bruit placentaire" by the French authors, and "placental-gerausch" by the Germans. Other authors, unwilling to involve any hypothesis in a name, prefer to call it "pulsation avec souffle," or "battement simple avec souffle." Ritgen calls it the "great beat," and he particularly alludes to the resemblance of this sound, with that which is usually audible in aneurismatic swellings, and more especially in that variety of aneurism, in which there is a direct communication between an artery and a vein. The extent, over which the placentary sound is usually to be heard, does not exceed a circle of an inch and a half, or two inches in diameter. Between each placentary bruit, there is no complete intermission of sound; for in the intervals there may generally be heard an obscure continuous sound, similar to, but much weaker, than the proper placentary bruit. At the corresponding part of the opposite side of the hypogastric region where the placentary bruit is most distinct, a similar and synchronous sound is sometimes perceptible. This arises no doubt from the mere resonance of the chief bruit; but in order that this resonance may be sufficiently strong to be perceptible, it is necessary that the uterus be well distended with the liquor amnii, and that its walls be firm and perfectly healthy. [Dr. Hohl assures us that, in some cases, he has distinctly heard the fluctuation of the liquor amnii. He observed that this latter sound was always most distinct just before any movement of the fœtus, or any contraction of the uterus.] The placentary bruit not unfrequently becomes all of a sudden much less audible than it had been the minute before.—Any change of position on the part of the woman, or any movement of the child will occasion this.

The second sound, peculiar to the state of pregnancy, is so remarkable, that if once heard it cannot possibly be mistaken for any other. As we have already said, it most nearly resembles the tic-tac of a watch, heard at some distance. The number of beats in a minute usually varies, from 108 to 170 or 180. It is observed to be quickened after any movement of the child, and when this ceases, it gradually resumes its former frequency. At other times it suddenly ceases, or at least is no longer audible; and then it as suddenly returns. It is quite independent of the maternal pulse, and appears not to be affected by any variations of it. With respect to the most common *seats* of the placentary and fœtal sounds, Dr. H. says that he has usually detected the former in the right epigastric region,* corresponding to the upper and posterior part of the uterus. In first pregnancies, it is almost invariably at this part that the placentary sound is heard. In subsequent pregnancies, it is not unfrequently heard lower down, and occasionally on the left side. As pregnancy advances, the seat of the placentary sound becomes gradually higher and higher in the abdomen.

The fœtal sound is most commonly heard in the left epigastric or hypogastric region.† Sometimes, but rarely, it is heard on the right side; and, more rarely still, in the course of the linea alba. As a general remark, we may affirm that the placentary sound is heard on the right side, and the fœtal sound on the left side. Sometimes, however, the very reverse is the case; but this is certainly of rare occurrence. Occasionally, both sounds are heard on the same side; and then the placentary is almost always seated higher up than the fœtal sound. Exceptions, however, to this statement have been detected by Dr. H.

Dr. H. has never been able to detect either the placentary or the fœtal sounds, by applying the ear to the back of the mother.

In reference to the periods of pregnancy at which the two sounds are usually first perceptible, Dr. Hohl's experience coincides with that of most of his predecessors. He has never heard the placentary sound before the fourth month, but frequently in the course of this month. At this early period, it is commonly heard in the right hypogastric region, obscure, and diffused over a considerable extent, without being decidedly more distinct at any one particular point. It is during the following, or fifth month, that it seems to become, as it were concentrated, or to be more loud and distinct at one point. Dr. H. has more than once observed, that the placentary sound became gradually more obscure as pregnancy advanced, without ever ceasing altogether; but, as a general remark, it may be affirmed that, as pregnancy advances, the placentary murmur becomes stronger and more extended, until towards the end of the ninth month, when it seems to be confined to rather a more limited extent than it was for some time previous. The fœtal sound he has never detected before the end of the fourth month, and rarely so early as this. In the fifth month, it is still very weak and indistinct, and it is only towards the middle or end of the sixth month that it is clearly recognizable. As pregnancy advances, it becomes more regular, and rather slower, than in the earlier months.

It is unnecessary to follow our author in the detail of numerous experiments, to prove that the placentary sound is invariably and uniformly synchronous with the maternal pulse, and, therefore, that it is affected by all changes in the frequency and force of this pulse. The fœtal sound, on the other hand, is quite independent of all these changes. The strength, indeed, of the fœtal pulse seems to be impaired, if at any time the respiration of the mother is impeded or distressed.

Dr. H. has never been able to satisfy himself whether the fœtal pulse is altered by any mental emotions affecting the mother. He is of opinion that its strength, but not its frequency, may be affected by such a cause.

With respect to the influence of diseases of the maternal frame on the fœ-

* This statement is surely not quite correct. The words of the author are, "in der rechtem oberbauchgegend," which are translated literally in the text; but we think that he must rather mean the upper part of the right hypogastric region; unless, indeed, his remark is intended to apply to the more advanced stages of pregnancy.

† These two regions—the epigastric and hypogastric, are far apart from each other. The vagueness of our author's language justifies the remark in the preceding note.

tal pulse, Dr. H. found that those, which are attended with any disturbance of the breathing, had the most marked effect. When the respiration, from any cause, was much impeded, the fœtal pulse usually became more and more feeble, until, at length, it could be no longer heard, In other diseased states, provided the breathing was not much affected, it seemed to be but little, if at all, affected. Dr. H. says that female fœtuses are less affected by dyspnœa, on the part of the mother, than male ones (!) He mentions a case where a woman, in a state of partial derangement, had made several attempts to hang herself, and, failing in these, had thrown herself into a pool. Fortunately she was rescued; but it was with extreme difficulty that she was resuscitated. In the course of the following night, she gave birth to a living female child. Large losses of blood render the fœtal pulse feeble and intermitting, or even altogether inaudible.

During the prevalence of the cholera, Dr. H. availed himself of several opportunities to ascertain the condition of the uterine and fœtal circulations, in patients labouring under this frightful malady. When the attack was not very severe, or that form of the disease, to which the name of cholerine was applied, existed, Dr. H. found that the placentary murmur was more feeble and indistinct; and that the fœtal pulse was but little affected, either in frequency or strength. But, in the more severe cases, where the cramps were severe, and the general exhaustion was great, the following changes were remarked by our author in four cases of pregnancy, which had advanced, in three, to the fifth, and in one, to the sixth month. In one case, with the sinking, or even the complete cessation of the radial pulse, the placentary murmur was found, at first to be rather increased than diminished in distinctness, and no change in the fœtal pulse was perceptible. Soon, however, the former sound became less and less strong, without entirely disappearing. Dr. H. was much in the habit of employing the affusion of cold water on the chest, with the view of relieving the oppressed breathing and circulation in cholera; and he tells us that so powerful was this simple remedy, that he repeatedly found that the blood, which before scarcely trickled from an opened vein, flowed freely, if the cold douche was used at the same time. This remedy had a very marked effect, in the present case, on the placentary murmur, rendering it at once more powerful and distinct. The fœtal pulse, all this time, remained but little affected. The woman recovered, and carried her child to the full time.

In another case, however, Dr. H. found that the placentary murmur became less and less obvious, as the pulse at the wrist sank. The fœtal pulse also became more rapid and indistinct, until it gradually vanished altogether. Delivery commenced and proceeded without almost any pains, and a dead fœtus was expelled, the woman seeming to be unconscious of what had taken place.

In a third case, the woman, 30 years of age, was seized with genuine cholera on the 30th of January. The placentary murmur was almost immediately affected, and the fœtal pulse became feebler and more rapid. On the following morning, the fœtal pulse was no longer perceptible. In the course of the afternoon, after a fit of violent shivering, labour commenced. The vagina felt, on examination, very hot, and the lower segment of the relaxed uterus, enveloping the head of the fœtus, was found to have descended low into the pelvis. The os uteri felt to the finger like a piece of soft wax. The pains of labour were very feeble. After three hours' continuance, a dead child was expelled, and the placenta followed immediately afterwards. Scarcely one drop of blood was lost; but a greenish-coloured gelatinous discharge escaped when the uterus contracted, after expelling its contents. The woman felt herself immediately afterwards better, and the remedies which were used against the cholera symptoms acted now with more decided benefit. The mammæ, which had become much shrunk, increased in volume, and the health of this patient was gradually but completely restored.

Dr. H. has had occasion to deliver several children, either by turning and extraction per vaginam, or by performing the Cæsarian operation, immediately after the death of the mothers from cholera—but in none of these cases did the children exhibit any traces of life. They, as well as the placenta and the umbilical cord, were always exceedingly pale and bloodless. The temperature of the interior of the vagina, and of the uterus, was in every case remarked to be extremely high. Dr. H. has had no opportunity of ascertaining the effects of syncope in the mother on the fœtal pulse.

In two cases, where regular menstruation continued until the sixth month of pregnancy, Dr. H. ascertained that, during the period of the flow, the placentary murmur was louder and more whizzing than at other times. This increase of sound is probably attributable to the increase of congestion in the uterine vessels, just before and during the discharge of the catamenia. We have already remarked, that the placentary murmur becomes more loud and distinct during labour-pains; and doubtless this is owing to the same cause. The fœtal pulse, in the two cases just alluded to, remained unaltered.

From the preceding observations, we may fairly conclude that the state of the circulation in the fœtus is in a very great measure independent of the maternal circulation, and that it is but little affected by diseases of the parent, unless these are accompanied with great dyspnœa, and obstruction to the pulmonary circulation.

### Changes in the two Sounds during Labour.

The placentary sound is much more uniformly affected by labour than the fœtal sound. It usually becomes more frequent, louder, and of a deeper tone during the whole process, and more especially just before the occurrence of each pain. Indeed, so very marked are these changes, that, by auscultation, we may discover the coming on and continuance of each pain, before the woman makes any complaint, and although she endeavours to smother it.

As soon as, or even before, each pain begins, we may hear a sudden and short continued rustling sound, which is probably occasioned by the agitation of the liquor amnii, in consequence of the contraction of the uterine walls, and of the movements of the fœtus. Almost at the same moment, the placentary murmur becomes louder and more distinct, and it is frequently accompanied with whistling singing sounds, not unlike those produced by striking a cord already in vibration, which previously were not perceptible. As the labour-pain increases in strength and extent, the placentary murmur seems to become gradually more and more distant from the ear, until at length it is extremely dull, or even altogether silent. As the pain reaches its acmé, and then gradually abates and ceases, so the bruit gradually returns, recovering the intensity which it had at the commencement of the pain, and at length resuming its original characters, viz. those which belonged to it before the setting in of the uterine contraction. These phenomena are not recognizable, if the pains be at all irregular; the increased distinctness of the sound, and its seeming removal to a greater distance from the ear, are then very partial and imperfect. The same remark holds true when the pains are excessively violent and short-continued. Indeed, the presence and the character of a labour-pain may be most truly ascertained by the experienced obstetrical auscultator, by his simply attending to the changes which happen to the placentary bruit, while the pain lasts, even although the woman should most heroically smother all expressions of her suffering. During the continuance of the pain, if the pulse at the wrist becomes fuller and quickened (and this is almost always the case), the placentary bruit will be found to be correspondingly affected, giving rise to what may be described as a regular ebbing and flowing of its intensity.

Dr. Hohl has had occasion, in several cases, to watch the effects of the secale cornutum on the uterine sounds, and he found that, very generally within a short time after its exhibition, the intensity of the placentary bruit was augmented, even before any increase of the pains was felt, or at least expressed, by the woman.

The effects of labour-pains on the fœtal pulsations are, on the whole, very inconsiderable. Immediately before the coming on of a pain, and just at the time when the rustling noise occasioned by the liquor amnii is perceived, the pulsation becomes rather less distinct, and seems as if it were further removed, or more distant from the ear, than it was before. But these changes are very slight, and soon pass away. They are no doubt attributable, in some degree, to the movements of the fœtus, and, therefore, they are not usually perceived when the head becomes fixed in the pelvis. When the liquor amnii has been discharged, and the child advances more and more to the outlet, the seat of the fœtal pulse becomes, as a matter of course, lower in the abdomen. When the pains are very severe, the fœtal pulse is less distinctly audible. Sometimes it ceases to be heard at all, even although the life of the child remains as perfect as ever.

Some difference of opinion has existed among authors, as to the real cause and origin of the sound to which we have, in the preceding pages, affixed the name of the placentary bruit, or placentary murmur.

This name, indeed, involves the hypothetical assumption, that the sound is owing to the passage of the blood into and through the substance of the placenta. Dr. Hohl adduces the following arguments in proof of this view of the question.

1. No such sound is heard in unimpregnated females. "For this murmuring pulsation (says he) is not a mere throbbing, as in the case of a large artery, nor a simple, one-toned beating, but a compound, many-toned, humming sound—sometimes sharper and higher, at other times deeper and more bass—now of a whistling character, and then having more of a hissing or whizzing one. These characters are, however, merely, as it were, accompaniments of the primary or essential bruit, and serve to prolong and to continue each cadence into the following one. This murmuring pulsation may, therefore, be described as consisting of one main or principal beat (haupt-schlag), accompanied with a resonance or after-sound, during the continuance of which another main beat recurs, so that there is never a complete interval of repose, or intermission of sound.

2. The murmuring pulsation (placentary bruit) may be heard in every pregnant woman, by a practised ear.

3. The sound becomes first audible when the uterine vessels enlarge, and prolong themselves to form the maternal portion of the placenta. By the fourth month, the placenta is developed, and it is usually about this period that the sound is first distinctly perceived. During the expulsion of the child, when the uterus has contracted upon itself considerably, and the close union between its walls and the adhering placenta is more or less interrupted, the sound becomes immediately more obscure. Even before labour has commenced, and, indeed, generally during the last week or two of gestation, the placentary murmur is less distinct than it had been, and it acquires, at the same time, a peculiar piping or whistling character. These modifications of the sound are, probably, owing to certain changes occurring in the placenta, and to a smaller quantity of blood being sent through it now than before. The impulse of the blood through the uterine vessels becomes less, in consequence of there being less occasion for the large supply of it towards the end of the ninth month; and, hence, the pulsatory murmur is not so strong as it had been previously. It is not easy to determine what is the cause of the piping or whistling notes, which so frequently accompany the sound in the latter weeks of pregnancy. Perhaps they are owing to the partial obstruction to the free transmission of the blood, in consequence of the uterine vessels becoming gradually more contracted, and to their being occasionally filled more or less with a calcareous deposit. Dr. H. says that, in those cases in which the whistling notes were heard by him most loud and distinct, the placenta was found, on expulsion, to be considerably altered and somewhat shrivelled (veralteste), as if it had been out of the body for some time.

4. The placentary murmur is heard much more frequently on the right than on the left side, and very generally opposite to the fundus of the uterus. Now it is acknowledged by all the best obstetrical writers, that by far the most common attachment of the placenta is to the fundus of the uterus, and oftener on the right than the left side. The reasonable inference therefore is, that the seat of the sound is connected with the position of the placenta.

Whenever the placenta is attached over the os uteri, the murmuring pulsation is invariably heard opposite to this part of the womb, and not in the usual place—higher up. The sound, too, is always more indistinct than under ordinary circumstances, in consequence, no doubt, of the greater depth in the abdomen of the cervix than of the fundus of the uterus, and also from the bloodvessels at the former part, never acquiring the same size that they do, at the latter.

5. Some auscultators having attributed the murmuring pulsation to the partial compression exercised by the pregnant uterus on the iliac bloodvessels, Dr. Hohl endeavoured to satisfy himself of the fallacy of this idea by the following experiments :—He examined several women in various attitudes—as standing, lying on the back, and leaning forwards, so that they rested the body on the knees and hands; thus the abdomen was protruded as much as possible, and in this way, any pressure of the womb on the arteries situated behind it was removed. Now, as in all these attitudes the murmuring pulsation was perceived, with almost equal distinctness, we may fairly conclude that it is independent of any bloodvessels merely adjacent, but not belonging, to the womb; and this inference is corroborated by the fact, that if we apply the stethoscope to the sides of the pelvis or abdomen while the woman is reclining forwards, we may often distinctly recognize the pulsations of the iliac arteries at the same time; but these pulsations will be found to be very different from the murmuring pulsation of which we are at present speaking.

6. The extent, over which the murmuring pulsation is usually audible, is limited to a space which nearly corresponds with the size of the existing placenta. In the earlier months of pregnancy, this extent is considerably smaller than in the more advanced stages; and Dr. Hohl has assured himself that, in the case of twins and triplets, the sound is generally extended over a much larger space than when there is only one fœtus present.

7. Dr. Hohl says that he could, with tolerable certainty, predict the presence of the calcareous deposition in the placenta (a state which is usually found in the placenta at the full period) by a certain piping or whistling tone, accompanying the usual placentary bruit.

We have already alluded to this accompanying sound, and have endeavoured to explain the origin of it, by supposing that some of the placentary bloodvessels become more and more obstructed by the calcareous matter deposited in their canals during the latter stage of pregnancy, and thus afford a certain degree of impediment to the passage of the blood through them.

8. When the placenta becomes, from any cause, partially detached from its connexion with the uterus, the distinctness, as well as the extent, of the placentary bruit is immediately diminished. When it is completely detached, the sound ceases entirely, " geht ganz verloren."

9. It is well known that some women suffer, during their pregnancy, a fixed pain at that part of the uterus, where it is generally believed that the placenta is attached. Now, in all the numerous examinations made by Dr. Hohl in such cases, he has invariably discovered the existence of the murmuring pulsation over that part; and he has also remarked that, when the navel-string has been coiled round the neck of the child, and the traction thus sometimes induced during labour has occasioned a pain at one part of the uterus, that part has always been at the very spot where the sound had been heard during pregnancy. Moreover, in cases where the placenta has adhered more firmly than usual after the expulsion of the child, upon dragging the cord with considerable force, so as to cause pain at the point of the uterus where the placenta was fixed, that point invariably corresponded with the seat of the placentary bruit.

10. M. Majon assures us that, if a fluid be injected into the umbilical cord, while the placenta is still adhering firmly to the uterus, the murmuring pulsation immediately becomes louder and more distinct than it was before, and we hear the same sort of streaming sound (fluth) as is perceived before the commencement of a labour-pain.

11. If the hand be introduced into the uterus after the expulsion of the child, the placenta will be found adhering to that part over which the murmuring pulsation had been heard.

From the arguments now adduced, it appears to us to be established, that the murmuring pulsation is owing to the current of the blood passing along the enlarged uterine vessels into the cellular substance of the placenta. The humming or gently buzzing sound, between each pulsation, may be ascribed, says Dr. Hohl, partly to the momentary tarrying or retardation of the blood during the interval between the pulsations—partly to the tremulous or oscillatory movements of the veins, and partly, also, to the electrical play of the globules of blood attracting, repelling, and blending with, each other in the cells of the placenta. This humming sound ceases almost entirely, when the communication between the substance of the placenta and the uterine parietes is interrupted.

The increase of the murmuring pulsation immediately before, and its diminution during, a labour-pain, are attributable, no doubt, the former to the accelerated, and the latter to the obstructed, circulation along the uterine and placentary vessels, in these two different conditions. If it be asked, how comes it that the murmuring pulsation is limited to the immediate seat of the placenta, and is not heard over every part of the uterus, seeing that all its bloodvessels are greatly enlarged during pregnancy, we answer, that the increase of the size of the bloodvessels in the neighbourhood of the placenta, is much greater than elsewhere; and, moreover, that the peculiar mode of the circulation from the uterine parietes into the cells of the placenta, is to be taken into account. The pulsatory murmur continues to be heard, but only very faintly, after the expulsion of the child, until the uterus has fairly contracted; and then it is no longer audible.

In a former part of this article, we have alluded to the marked resemblance of the pulsatory murmur with the humming sound which follows it, to the auscultatory phenomena of a large aneurismal varix. Dr. Hohl considers that the structure of the placenta is similar to that of such a varix; "for here (says he) there is the same mixing of the arterial with the venous blood, the same friction of the blood-globules upon each other, the same attracting and repelling between them, and the same electrical play to which the blood in the placentary cells is subjected."

Our author having discussed the subject of the murmuring pulsation or the placentary bruit, proceeds, with his accustomed wearisome minuteness, to expound the reasons for his belief, that the other auscultatory phenomenon peculiar to, and indicative of pregnancy, is owing to the action of the fœtal heart.

It is almost unnecessary to follow him through these details, as we suppose that, in the present day, no one is inclined to question the correctness of this explanation.

The reason of this sound being not audible before the fifth month is doubtless the small size and feeble action of the fœtal heart. We may also add, that it is during this, the fifth month, that the fœtal heart is directed towards the left side, and that the arterial or left half of the organ, hitherto much smaller than the venous or right half, becomes larger, and attains that proportionate size which it retains ever afterwards. The fœtal sound is invariably most distinctly heard over that part, where the left side of the fœtal thorax is lying. When the uterus is more than ordinarily distended with liquor amnii, the sound of the fœtal heart is heard over a larger extent than usual. As a very general rule, the fœtal sound is heard on the side opposite to that in which the placentary bruit is audible. In the case of twins, the tic-taccing sound may frequently be heard in two different places, provided the fœtuses be quite motionless at the time of examination. In the case of triplets, Dr. Hohl tells us that it is not possible to distinguish the various pulsations; "all is a chaotic beating; and the difficulty is increased by the loudness of the placentary murmuring under such circumstances."

Whenever the umbilical cord protrudes through the os uteri into the vagina before the child, and we are enabled to count its pulsations, the number of these will be invariably found to correspond with the number of the beats of the fœtal heart heard through the abdomen of the mother; thus establishing, beyond all controversy, the true nature and origin of these sounds.

The younger the fœtus, the more frequent is the pulsation of the heart. The frequency of the pulsations is always greater after any movements of the fœtus. This may be very easily ascertained, at any time, by merely pressing the end of the stethoscope rather firmly against the spot where the fœtal pulse is audible. A rise of 20 or 30 beats is then often almost immediately perceived.

The following tables will illustrate this fact, and exhibit, at the same time, some of the changes which the pulse undergoes immediately after the child is born, and when respiration has commenced.

| No. 1. | *Pulse.* | *State of the Child.* | |
|---|---|---|---|
| 27th July | 115—140 | Quiet | In utero. |
| ——— | 156 ——— | After movement | In utero. |
| 30th July | 108—140 | Quiet | In utero. |
| ——— | 158 ——— | After movement | In utero. |
| 31st July | 120—125 | During the 2nd period of labour | |
| ——— | 112 ——— | During the 3d period | |
| ——— | 108 ——— | Immediately after birth | |
| ——— | 104 ——— | 12 hours after birth | |
| ——— | 126 ——— | 24 do. do. | |
| ——— | 146 ——— | 48 do. do. | |
| **No. 2.** | | | |
| 21st Jan. | 86—120 | Quiet | In utero. |
| ——— | 140 ——— | Restless | In utero. |
| 22d Jan. | 110—120 | Quiet | In utero. |
| ——— | 136 ——— | Restless | In utero. |
| ——— | 108 ——— | During the 2nd period of labour | |
| ——— | 108 ——— | During the 3d period | |
| ——— | 100 ——— | Immediately after birth | |
| ——— | 108 ——— | 12 hours after birth | |
| ——— | 110 ——— | 36 do. do. | |
| ——— | 140 ——— | Fifth day do. | |
| **No. 3.** | | | |
| 1st Feb. | 140 ——— | Restless | In utero. |
| ——— | 88—136 | Quiet | In utero. |
| 2d Feb. | 90—136 | Quiet | In utero. |
| ——— | 140—150 | During the 1st period of labour | |
| ——— | 130—138 | During the 2d period | |
| 3d Feb. | 108 ——— | 12 hours after birth | |
| ——— | 100 ——— | 24 do. do. | |
| ——— | 140 ——— | 36 do. do. | |
| ——— | 88 ——— | 42 do. do. | |

| | Pulse. | State of the Child. |
|---|---|---|
| **No. 4.** | | |
| 22d Jan. | 144—156 | Restless } In utero. |
| ——— | 108—112 | Quiet |
| 23d Jan. | 154—165 | Restless |
| ——— | 110—120 | Quiet |
| 24th Jan. | 140 —— | Quiet |
| ——— | 160 —— | Restless |
| ——— | 104—148 | During 2nd & 3d periods of labour |
| ——— | 146 —— | Immediately after birth |
| ——— | 140 —— | 12 hours after birth |
| ——— | 154 —— | 24 do. do. |
| ——— | 140 —— | 36 do. do. |
| ——— | 108 —— | 48 do. do. |
| ——— | 140 —— | 72 do. do. |
| ——— | 170 —— | Fifth day do. |
| **No. 5.** | | |
| 5th May | 108—154 | (Not stated) } In utero. |
| 7th May | 110—148 | Quiet |
| 8th May | 130—140 | During delivery |
| ——— | 130 —— | Immediately after birth |
| ——— | 150 —— | 24 hours after birth |
| ——— | 154 —— | 36 do. do. (restless) |
| ——— | 170 —— | 60 do. do. (after crying) |
| ——— | 108 —— | 72 do. do. (asleep) |
| **No 6.** | | |
| 12th May | 106—130 | Quiet } In utero. |
| ——— | 154 —— | Restless |
| 14th May | 130—140 | During labour |
| ——— | 156 —— | Immediately after birth |
| 19th May | 108—140 | Asleep—restless. |

*Die Geburtshülfliche Exploration, von Dr. Hohl. Halle,* 1833.

---

### Cases of a Milky State of the Blood.

A man, 47 years of age, much addicted to excess in wine, was admitted into the Hospital at Cremona with the symptoms of pneumonia, attended with high feverish excitement. He was freely bled and purged. On examining the blood on the following day, it was found to be quite white—the clot as well as the serum, and it exhibited a milky smell. The urine, too, which was secreted in very small quantity, had a milky appearance; and the whole surface of the body, and more especially the face, exhibited an extreme pallor. The bleeding was repeated on the following two days, and the blood presented the same phenomena. On the 26th (the 5th day after his admission), the symptoms, although still severe, were considerably abated; a fresh venesection was however ordered. The crassamentum of the blood presented a more normal appearance, but it was covered with a thick buffy coat: the serum was white and "n'offrait point les principes qui constituent le serum." On the following day, he was again bled; and the blood drawn was almost natural in appearance, the serum being yellowish, and the clot free from buffiness. After this date, the patient speedily recovered.

*Chemical Analysis of the Blood.*—Having decanted off the serum along with the clot, a whitish turbid fluid, having many of the characters of milk, and exhaling the smell of butter, remained at the bottom of the vessel. A portion of the buffy coat, which was at least half an inch in thickness, and had a distinct greasy smell, having been immersed in boiling alcohol, furnished "une substance grasse" similar to what was obtained from the serum, to be immediately described. Mixed with the crassamentum were drops of an oily liquid, having "tous les caracteres exterieurs de lait." The fluid obtained by pouring boiling alcohol on the serum, and filtering the mixture, yielded on evaporation a flocculent fatty matter, similar to the crystallizable matter of M. Lecanu, or to the seroline of M. Baudet. The albuminous matter of the serum remaining on the filter "etait fort onctueuse," and when digested with repeated quantities of boiling alcohol afforded a fatty oleaginous substance, of a yellowish colour, soluble in alcohol and æther, forming an emulsion with water, and when left exposed for some time, becoming acid and emitting a fatty smell. The albumen was obtained pure, by digesting it subsequently in æther. No traces of the proper sugar of milk could be detected.

From these results, it appears that this fatty substance in the blood was similar to what exists in chyle, and also in several respects to cerebral matter. 1000 parts of the serum consisted of 905 of water; 76 of albumen; 4 of crystallizable fatty matter; 6 of oleaginous matter; 5 of extractive, lactate of soda, chloruret of sodium, and chloruret of potassium; and 4 of other salts.—*Annali Universali.*

A case very similar in many respects to the preceding one occurred in our practice about a twelvemonth ago. The patient, a woman, 33 years of age, and whose appearance strongly indicated a tuberculous constitution, was seized with pleurisy and pericarditis. She was bled for six days successively; and each quantity of blood was most remarkable for the very great thickness of the buffy coat (half and sometimes two thirds of an inch thick) and for the strikingly milky appearance of the serum. We had never seen these phenomena to so great an extent before. As to the cause of the milkiness of the serum, perhaps the opinion which attributes it to the presence of unchanged chyle in the blood is the most probable. The experiments of Christison, Babington and Lecanu agree with those of the Italian author, that, in this kind of blood, an unusual quantity of oily matter is discoverable in it. What are the states of the system in which the blood is apt to exhibit a milky appearance? Our experience prompts us to answer, the existence of active inflammation, especially of the respiratory organs. Whether there is necessarily any coexistent affection of the thoracic duct we are not prepared to say. Dr. Copland in his Medical Dictionary has stated "that the milky state of the serum is occasionally met with in various cases, functional as well as organic; and seems connected with deficient assimilating powers."

The following case is reported in the number of the French Lancet for April last. A man 48 years of age labouring under extreme dyspnœa, was admitted into the Hôtel Dieu: the skin was hot and dry, the movements of the heart rapid and tumultuous, and auscultation indicated that there was a great obstruction to the free ingress of air into the lungs. A vein in the arm was immediately opened; but "judge," says the reporter, "my surprise at seeing blood as white as milk, stream from the orifice."

Twenty ounces of this white blood were withdrawn. In the evening leeches were applied to the epigastrium, and the blood which flowed from the bites exhibited the same appearances as that obtained from the arm in the morning. (We are not supplied with the report of the rest of the case, and are informed only that in the course of a week the patient had recovered his health.)

The blood was analysed by M. Lecanu. Its appearance, says this distinguished chemist, was so peculiar, that I cannot describe it better than by comparing it to "une bavaroise (tea sweetened with capillaire) legerement rosée." On resting, it deposited traces of colouring matter, the fibrine of which seemed to have disappeared ; and above this deposit, there was found a white opaque fluid, like milk or a strong emulsion.

In a 1000 parts of the blood, there were 794 of water ; 65 of albumen ; 108 of fatty matter, acid soap (savon acide) and cholesterine ; 17 of oleine, margarine and stearine ; and 25 of salts and extractive matter.

The fibrine and especially the colouring matter of the blood had almost entirely disappeared, and the place of the globules was supplied with a corresponding quantity of fatty matters, of which the acid matter and the cholesterine belong to healthy blood, but not so the oleine, margarine and stearine. It is therefore to the presence in large quantities of these fatty matters, held in suspension in the serum, that we must attribute the peculiar appearance of the blood in this case.

---

### On the Chemical Condition of the Saliva, as an Indication of the different Morbid Affections of the Stomach.

M. Donné is well known in Paris as one of the most zealous and intelligent young physicians there. For several years he was clinical clerk to M. Louis, as our readers may have learned from the foreign Periscope of our Number for July last; and subsequently he attached himself very enthusiastically to the service of M. Bouillaud in the wards of the great Hospital La Charité.

The pages of the Journal Hebdomadaire for the last three years attest his great activity as a diligent and enlightened observer of disease. For some time past he has been engaged in collecting materials for a work on the Chemical Properties of the Animal Fluids in health, and also during the progress of various morbid affections.

One branch of the subject appertains to the investigation of the changes which the saliva undergoes in disease, and more especially in that form of it, which has been of late years far too indiscriminately designated gastro-enteritis. Although a devoted disciple and admirer of M. Bouillaud, (who is generally considered as one of the most ardent and able followers of the physiological or Broussaian doctrines,) he candidly admits that a multitude of stomach affections, differing greatly in their characters, and requiring very different modes of treatment, have been most erroneously grouped together under this much abused epithet. Numerous are the cases, where patients have been tortured with the long continued but ineffectual use of antiphlogistics and refrigerants, and where a few doses of emetics and purgatives subsequently have dissipated all distress in the course of a day, or two. The diagnostic symptoms between the genuine " gastro-enterite," and mere " embarras gastrique," hitherto pointed out by medical writers are indeed, it must be confessed, often unsatisfactory and even delusive ; and it has been owing in a great measure to this very uncertainty that the exclusive doctrines first promulgated by Broussais have been carried to so extravagant a pitch by one party, and condemned so indiscriminately by another.

It is with the hope of dispelling part at least of this uncertainty that M. Donné has ingeniously proposed the chemical state of the saliva as a means to determine the real character of various stomachic diseases.

He premises his remarks by alluding to those excellent observations of M. Andral, wherein he states " that no relation can be established between the existence of nausea and vomiting, occurring during the progress of fevers, and any lesion of the stomach appreciable on dissection ; for, on the one hand, these symptoms have been often absent, when the stomach has been found ' le plus rouge et le plus gravement affecté ;' and on the other hand, when they have been present, often no morbid change of the viscus is discoverable after death. The mere existence therefore of these symptoms in fevers is no proof of even irritation, far less of decided inflammation, of the stomach.

Unless the vomiting be accompanied with epigastric pain, redness of the tongue and thirst, we have no good reason to predicate the existence of gastritis. What M. Andral says of the state of the stomach in continued fevers, is equally applicable to many other cases of less serious importance, where the physician is not a little perplexed to determine whether there exists a mere "embarras gastrique" (in the language of Stoll "saburres gastriques") or a decidedly irritative or inflammatory condition of the stomach. In alluding to the treatment he adds: "unfortunately there must always remain some uncertainty as to the actual morbid state of the stomach in those cases, where the symptoms have been relieved by the use of emetics and purgatives: and to add to the difficulty the symptoms which have been supposed to indicate the use of such remedies, are by no means uniform and constant." The object of M. Donné's Essay is to endeavour to remove some of the difficulties, which environ this interesting department of Semeiology, and to point out a method by which the physician may be enabled to ascertain the various "modifications morbides," in which the employment of emetics is or is not appropriate: and first with respect to the inflammatory irritation of the stomach he directs our attention to a symptom hitherto unnoticed by medical men, and derivable from the chemical state of the saliva. Before alluding to the pathological modifications of this fluid, it may be useful to glance at the opinions of different authors as to its chemical properties in a state of health.

M. Magendie regards the saliva as being "sometimes acid, sometimes neutral, and sometimes strongly alcaline: when the stomach is empty it is usually acid; during mastication it is alcaline, the acidity disappearing sometimes dès la premiere bouchée d'alimens." (A tolerably fair specimen of the vagueness of this physiologist's assertions.)

According to M. M. Tiedemann and Gmelin, the saliva and the mucus of the œsophagus are alcaline in all cases, not only in man, but in every other animal, which they have examined.

M. Donné's experiments have led him to the same conclusion. He has found that the saliva is constantly alcaline before, as well as during, taking food, and also during digestion, provided the stomach is in a healthy condition. He assumes it therefore as a position beyond all doubt that the normal state of the saliva is decidedly alcaline. Any one may readily satisfy himself of its truth by merely moistening with the saliva a slip of litmus paper which has been reddened by a weak acid: the blue colour of the dye is quickly restored. The saline contents are chiefly lactate and muriate of soda, and muriate of ammonia.

It is quite true that the saliva will be found not unfrequently to be acid; but this change of chemical property is always coincident, according to our author's experience, with a morbid state of the stomach; and it is on this very change of character that M. Donné founds the diagnosis of several diseases of this organ. He repeats the assertion, that he has never met with a single instance of the saliva being acid, when the functions of the stomach were healthily performed; and he assures us that whenever it is so, he has always found it to be characteristic of irritation or of inflammation of the mucous coat.

Hitherto there have been only two methods by which the existence of such a morbid state in doubtful cases has been deducible; viz. either by attention to the effects of the remedies, antiphlogistics or tonics, refrigerants or irritants, sedatives or evacuants, which may have been administered; or secondly by post mortem examinations. If the axiom "naturam morborum ostendit curatio" be not always rigorously true, it must be admitted that its principle has guided and still guides many excellent physicians, and serves as a basis for most of the reasonings which we daily make in appreciating the value of different therapeutic means. We are willing to admit that it is an essentially empirical and often a misleading guide; but we are not to reject its assistance merely from the abuses which some have made of it. Medicine is in an especial degree a science of minute observation; and our observations to be practically useful are not to be confined to one set of phenomena to the exclusion of any other. Morbid anatomy acquaints us with one set; semeiology, or the study of symptoms teaches us another; and therapeia or the doctrine of the action of remedies furnishes us with a third.

The chemical examination of the secretions during life belongs to the second of these great branches of medical science. Hitherto it has been far too much neglected; for, with the exception of urinary complaints, there is scarcely a class of diseases, in which its assistance has ever been invoked.

Without further details, we shall now narrate briefly a few cases which will illustrate the good effects of attending to the chemical properties of the saliva in disease.

It may be necessary to state, that the only testing means, which M. Donné recommends, is the use of slips of litmus paper, such as are generally employed in examining the urine. Some of them are to be reddened by immersion in a weak acid, to serve as tests of alcalinity.

The saliva of a man, labouring under intense bronchitis, was uniformly found to be acid. On dissection, the mucous membrane of the stomach was found "pointillée et ramollie" in some points, and red and highly injected in others.

A young woman, when admitted into La Charité Hospital, was labouring under a severe attack of bronchitis, attended with great tenderness of the abdomen, excessive irritability of the stomach, diarrhœa, ardent thirst, &c. The saliva was strongly *acid*.

The disease assumed in its progress a marked typhoid character, the tongue was parched and coated with a brown crust; the abdomen was always very tender; delirium and coma supervened, and the patient died on the 10th day after admission. The saliva was acid during the whole course of the illness.

*Dissection.* The mucous coat of the duodenum and jejunum was highly injected; the intestinal follicles were enlarged, and many of them ulcerated. The stomach exhibited "une injection pointillée" in its large extremity, and its mucous coat was soft and easily lacerable in several places: in other places it was very vascular, and of a dark colour.

A young man was received into the La Charité Hospital as a fever patient. All the symptoms of ataxic fever soon developed themselves. The saliva during the first days was only slightly acid; but later it became more strongly so. He died comatose. The saliva remained acid to the end.

*Dissection.* The mucous coat of the stomach was healthy in four-fifths of its extent; its large extremity, however, exhibited "une rougeur pointillée avec ramollissement pulpense de cette membrane."

In a patient who died of severe pneumonia, complicated with great irritability of the stomach and with diarrhœa, the saliva had been found to be unusually acid, so that it reddened the litmus paper "comme le ferait du vinaigre." Besides serious lesions of the thoracic viscera, the inner surface of the stomach presented in different places a state of high injection, of partial softening, and of complete disorganization of the mucous tissue.

The preceding cases are intended to illustrate the coincidence between a permanently acid state of the saliva and the existence of inflammatory irritation of the stomach, as ascertained by dissection. We shall now detail some cases, where the saliva was acid at first, but gradually lost its acidity and recovered its alcaline properties, as the patients were restored to health.

A young man was admitted into the hospital as a fever-patient. The symptoms were not serious: there was a yellow hue of the skin; the epigastrium was rather tender on pressure; there were however neither vomiting nor diarrhœa present; the tongue was white, and the saliva was alcaline. During the progress of the case the saliva became acid, continued to be so for three days, then became neutral, and as the patient recovered, gradually resumed its alcalinity.

A student of medicine, who had been but scantily fed for several months, was seized with shivering, pains in the loins, loss of appetite, &c. The epigastrium was tender on pressure; the tongue was covered with a thick white coating; the saliva was strongly acid. As the feverish symptoms subsided, the saliva became neutral, and during convalescence, it recovered its alcaline properties.

A young woman was attacked with the symptoms of gastro-enteritis. The saliva was decidedly acid. Antiphlogistic remedies were used with happy effects, and the saliva gradually shewed itself less and less acid, until it became neutral, and at length normally alcaline.

In the case of a young man, who exhibited the symptoms of gastritis, viz. great tenderness of the epigastrium, thirst, tongue red and parched, &c. the saliva was found to be decidedly acid. By repeated leechings of the abdomen, and the use of demulcent and refrigerant drinks, the symptoms were speedily relieved, and in a few days the saliva was quite neutral, having no effect either on the simple litmus paper, or on that which had been previously reddened by an acid. It soon regained its alcalinity.

This patient had two relapses of the complaint; and on both occasions the saliva was acid at first, and became neutral and then alcaline, as the symptoms disappeared

From the preceding data, M. Donné deduces the important (if verified by future enquiries) conclusion, that in all cases of gastric disturbance, when the saliva is distinctly acid, we have reason to believe that an inflammatory irritation of the stomach is present, and therefore that an antiphlogistic treatment is required.

With respect to the converse of the proposition, he candidly admits that his facts are not yet sufficiently numerous and definite to warrant him in laying down any therapeutic indication; and he only *suggests* that, whenever the saliva remains alcaline in gastric complaints, the most appropriate remedial means will be found to be emetics and purgatives. He promises to continue his own researches, and invites others to follow the same path of inquiry.—*Archives Génerales.*

---

Small-pox at Naples.—Dr. Gregory and others on the late Epidemic in London.—Reciprocal Effects of Variola and Vaccinia.

From the beginning of May to the middle of October, 1834, the small-pox was very prevalent in Naples. The epidemic was more severe than had been witnessed for the preceding thirty years. The number of patients affected with it was upwards of 7000, and of these 1450 died. It appears that the prejudices and culpable negligence of the lower orders, in regard to vaccination, are quite as common at Naples as elsewhere; for we find that out of 15,000 children born there annually, 6000 at least are never vaccinated.

The greatest mortality occurred in the districts Mercato, Pendino, and Porto; and more especially at Aversa and Afragola, where one case in every three proved fatal. Persons of all ages, who had been effectually vaccinated, seldom or never caught the infection. Many of them, indeed, were seized with varicella; some with febrile pemphigus, and others with miliaria, urticaria, or other eruptive diseases, but not one exhibited the genuine variolous exanthema.

Several cases of the variolous and the vaccine eruption being coexistent were observed. If the vaccine pustule was established before the development of the variolous disease, and if it proceeded regularly through its various stages, the variola was always observed to be considerably modified, and invariably favorable in its event. But if, on the other hand, the variolous eruption was copious and fully formed, the vaccine pustule became smaller, and, at it were, withered, and it speedily dried off. The conclusion, therefore, seems to be, that whichever disease affects the system primarily, and antecedently to the other, prevents the full development of the latter. In the late epidemic at Naples, the eruption was very generally confluent. The attendant pyrexia usually assumed the form of a continued gastric fever; sometimes it was truly inflammatory, and at other times, but more rarely, it was nervous or putrid. The greater number of the cases occurred in young children. Both sexes were almost equally affected; but the greatest mortality was among the female patients.

One example of a second attack of the disease was observed at Aversa. A young man, who had suffered from variola during his infancy, and who still retained the marks of it, was seized; but he soon recovered.

M. Ronchi, the author of these observations, and president of the Central Board of Vaccination at Naples, repeats the assertion, that in the numerous cases which fell under his notice, not one well-marked instance occurred in a patient, who had been duly vaccinated, and who bore the genuine marks of the operation on his arm. But it appears that he witnessed numerous examples of confluent and very severe consecutive varicella, which other authors would doubtless have considered as secondary or modified variola. It is therefore rather an apparent than an actual difference of opinion, as to the immunity of vaccinated persons from an attack of small-pox; because what some would regard as mere varicella, others would consider to be variola in a modified form. The species of varicella, which Signor R. describes as having been most prevalent, was the v. globulosa of Bateman, the v. pustuleuse globulaire of Alibert. He confesses that the diagnosis between this form of varicella and the modified variola, was sometimes very difficult; but that almost always the peculiar shape of the pustules, the absence of the central depression, the larger quantity of their contents, and the more rapid succession of their phases, were sufficient to announce the varicellar character. The treatment generally adopted was very simple. When the eruption was distinct, and the pyrexial symptoms were not very severe, the use of cooling subacid drinks, and of mild farinaceous food, the proper ventilation of the sick-rooms, and the occasional administration of gentle aperient lavements, were all that was necessary. The truth of Ramazzini's remark, " facilius evaserunt ei, quibus nec detractus fuerit sanguis, nec ullum administratum remedium, toto curationis negotio naturæ commisso," was amply confirmed by Signor R.'s experience in the late epidemic. When the feverish symptoms ran high, especially in young and plethoric subjects, and when the head suffered from congestion, a bleeding from the arm was usually practised. Except in those cases, and they were rare, bleeding was seldom resorted to, and never with any but hurtful effects.—Mild expectorants, such as the spiritus Mindereri and vinum ipecacuanhæ were useful. Purgatives were seldom employed before the stage of the desiccation of the pustules. When used earlier, they often interrupted the course of the eruption, and excited disagreeable nervous or gastric symptoms.—*Filiatre Sebezio di Napoli.*

The tendency which the small-pox has shewn of late years to re-assume its formidable ascendancy among epidemic disorders, and the irregularity not only of the protective influence, but also of the common phenomena, of the cow-pox, have excited the attention of medical men, in almost every country in Europe. Many districts of France have recently been visited by small-pox, and have suffered severely from it. The Prussian Government has been alarmed by its wide-spreading among their army; and several of the Italian states have appointed commissions to examine the histories of some late epidemics among them. Our own country, it is well known, has not escaped the ravages of an enemy, which, it was too fondly supposed, had been almost extirpated. " The snake was scotched, not killed." During 1834, the small-pox was unusually prevalent in London, and its suburbs. Dr. Gregory, physician of the Small-pox Hospital, whose opportunities of observation are so much more extensive than any private practitioner can enjoy, has done well to communicate the results of his inquiries to the profession. In a paper which he lately read at the College of Physicians, and which was published in the Medical Gazette, there are several interesting facts, which we shall now briefly enumerate. It appears from the report of the National Vaccine Establishment, and from the statements of Dr. G., that the variola of 1834 was not only less prevalent, but was also much more mild and favorable than it had been for many years preceding. The average rate of mortality had been previously about 30 per cent. In 1834 it fell from 30 to 14. " The small-pox of last year (1834)," says Dr. G. " may therefore be looked upon as the mildest ever known in this part of the world."

Towards the close of the year, the disease began to manifest greater severity, and speedily reached its average fatality, and up to the middle of summer, it had not very sensibly abated in malignancy. According to the researches of Dr. Gregory, it appears, that from the year 1826, to the current one, (1835,) the proportion of the small-pox cases occurring after vaccination to the entire number of persons affected, has been, on the whole, very steady and uniform; the ratio varying only from 32 to 38 per cent. The frequency, therefore, of the modified or varioloid disease, seems to be nearly correlative with the frequency, but not with the malignancy, of the small-pox itself. In 1828, when the rate of the mortality was so high as 33 per cent., the proportion of the unvaccinated cases, 73, to the whole number, 202, admitted into the Small-pox Hospital, was 36 per cent.; whereas, in 1834, when the rate of the mortality was only 14 per cent., the proportion of the unvaccinated cases, 63, to the whole number, 165, admitted, was as high as 38 per cent. The severity of the one epidemic and the mildness of the other appear to have had no connexion with, or dependence upon, the comparative rarity or frequency of the modified disease.

From the steadiness which the average number of the unvaccinated cases has annually borne to the aggregate of the admissions into the hospital, during a period of nearly ten years, Dr. Gregory is inclined to believe "*that we have now reached the maximum of vaccine imperfection;*" and "that we already know the full extent of the evil."

From Dr. G.'s calculations, we may infer that in the year 1834, 2386 persons laboured under small pox in London; and that of these, 906 had been previously vaccinated. The proportion of the vaccinated to the unvaccinated may, as we have already mentioned, be estimated at 34 or 35 per cent. This is indeed a much larger proportion than was ever anticipated in the days of Jenner; but it is worthy of notice, that the average mortality by small-pox in London has been nevertheless reduced three-fourths since the introduction of vaccination.

Dr. G. has endeavoured to ascertain the cause of the deterioration of the vaccine operation as a protecting means against the assault of the small-pox. He adverts to the possibility of the lymph having become in some degree *humanized*, or altered in its qualities, from having passed through so many human bodies since it was first derived from the cow. It appears that the lymph now in use in London, and throughout this country, was originally procured from Mr. Harrison's dairy in Gray's-Inn Lane in 1799, and that it has not been renewed since that period.

"The appearances on the arm," says Dr. G. "certainly favour the idea that no deterioration, or material alteration, in the qualities of vaccine lymph, has taken place since the commencement of this century;* but I have always felt, that to put this important point beyond the reach of doubt, it would be very desirable to have recourse occasionally to that experiment on which alone public confidence in vaccination was originally founded;—I mean variolous inoculation. The numerous experiments performed by the early vaccinators sufficiently prove that, when variolous inoculation is practised within a few weeks or months after vaccination, no effect, either local or constitutional, is produced; or, at farthest, a pimple only forms at the place of puncture, and this subsides in the course of two or three days, without having undergone any regular suppuration."—From some experiments made by Dr. Gregory in 1831, and repeated during the present year, for the purpose of ascertaining the effects of variolous inoculation at more remote periods from the date of the preceding vaccination, "it would appear that, when the interval from the date of vaccination exceeds twelve months, the vaccine lymph now in use does not prevent the usual *local*

* In our experience for the last four or five years, the development of the vaccine vesicle has been often very irregular; generally more tardy by two, three, or four days, than it used to be. —*Rev.*

effects of the variolous virus, but that it arrests the process of inoculation at that point when febrile commotion and constitutional eruption are wont to appear." The facts which have led him to this conclusion are the following. A child, four years of age, and who had been vaccinated when six months old, was inoculated with fresh variolous virus, by Dr. G. "On the sixth day, the arm presented the appearance of a regularly formed vesicle on an indurated base, and surrounded by an areola. No febrile disturbance or eruption however succeeded. In a few days the vesicle desiccated."

A young girl, ten years of age, was vaccinated in 1821, and in 1831 the variolous inoculation was performed by Dr. G. On the eighth day after the insertion of the virus there were pustules formed at the puncture, but neither febrile disturbance, nor any constitutional eruption succeeded.

The third case is especially worthy of notice.

Mary Ann Ward, aged two years and four months, vaccinated a year and a half previously, and who had been exposed, but with impunity, to the contagion of a very virulent small-pox, which proved fatal to the patient, and which had infected her younger brother, was inoculated with variolous matter on March 8th. "On the 11th the arm presented the most perfect appearances of a successful inoculation. On the 15th a perfect vesicle with depressed centre had formed, upon an indurated basis, with considerable surrounding inflammation, of irregular shape. No febrile commotion, however, nor any constitutional eruption occurred."

The history of the disease in this girl's brother is remarkable, as it shews that vaccination may advance and run its perfect course, while the system is incubating, and preparing to throw out perfect small-pox; and that under these circumstances, the lympth of the one vesicle will communicate genuine vaccinia, and the lympth of the other genuine variola. The parents of the children were living on the same floor with a man who had died of the small-pox on the 23d February; and both children had been frequently in the sick-room, both before and after the nature of the patient's complaint was known. On the day after his death, the youngest child, which was only four months old, was vaccinated. Nine incisions were made, every one of which took effect, and several children were successfully vaccinated with the lymph taken from the patient on the 3d of March. On the evening of this day, the infant sickened, and on the 5th a copious eruption of genuine variolous vesicles shewed itself over the whole body. At this time the vaccine vesicles on the arm were large and full, and surrounded by a fine and perfect circular areola. The small-pox advanced, and ran its course altogether unmodified. On the 17th, the small-pox pustules were drying over the whole body; the vaccine scabs on the arm also were drying, but were still adherent. It was with matter taken from this infant, on the fourth day of the variolous eruption, that the eldest child, his sister, whose case we have just narrated, was inoculated.

In some of the recent numbers of Rust's Magazine there are several valuable communications respecting the epidemics of variola, which have lately prevailed in different parts of Germany. The most important of these is from the pen of Dr. Ebers, physician to the General Hospital at Breslaw. The conclusions, which he has drawn from a very extensive experience, are highly satisfactory as to the protecting influence of vaccination. Indeed all the German physicians appear to be unanimous on this subject, that genuine variola is exceedingly rare in a person who has been duly vaccinated, and that a very large majority of the cases which have been reported as such, either have been examples of varicella rather than of variola, or, if they have been examples of the latter, that they have occurred in persons on whom vaccination had been imperfectly and improperly performed.

From December, 1831, to the middle of 1833, ninety cases of genuine variola were treated by Dr. E. Of this number, three only are recorded as having occurred in persons previously vaccinated; nine in persons, whose vaccination was very doubtful, and seventy-eight in unvaccinated persons.

As to the three cases first-mentioned, it is proper to observe that in two of them the patients exhibited no characteristic cicatrices on the arms: these, therefore, can scarcely be admitted as unobjectionable examples of the failure of the cow-pox protection. In all the cases enumerated as "doubtful," it could not be satisfactorily ascertained whether the patients had been vaccinated at all or not, in consequence sometimes of the want of authentic information as the operation having ever been performed, and at others, from the utter absence of all cicatrices on the arms.

Of the ninety cases, forty-eight proved fatal; one among the vaccinated, five among the doubtfully vaccinated, and forty-two among the unvaccinated individuals. The mortality was therefore very high, exceeding the proportion of one-half of the whole number. This was in a great measure attributable to the disease having very generally been neglected for several days before medical aid was summoned.

Of the varioloid or modified form of the disease, (whose distinctive characters are the great mildness of the eruptive fever, the irregularity of development and want of uniformity in the appearance of the eruption, the great rapidity of the pustular changes, the absence of the peculiar variolous smell from the surface, and also of the secondary fever, and the less depth and size of the cicatrices left behind,) there were 146 cases, of which 137 occurred in vaccinated persons, and nine in patients whose previous vaccination was doubtful. Only two of the entire number of these cases proved fatal; and in both of them it was "doubtful" whether the patients had been ever vaccinated.

Of varicella, 210 cases were seen by Dr. E. In all, the patients recovered. In two only was the vaccination of the patients doubtful; and in all the rest, it was distinctly and unequivocally proved. Surely no results can proclaim more satisfactorily the paramount utility of vaccination. Out of 146 cases of the different forms of "pocky disease," three only proved fatal to persons who had been previously vaccinated.

Dr. E. alludes to the frequent complaints which have been made within late years of the inefficiency of vaccination, as a means of protection against the attacks of small-pox, and he approves of having recourse to virus obtained directly from the cow. Nevertheless he is convinced from the results of his own experience, that the greater number of failures are attributable rather to the faulty and imperfect manner in which too often the operation has been performed, or to the negligence of the parents of the children not taking them to the surgeon afterwards, in order that the true nature of the pock might be ascertained, than to any well-established deterioration of the vaccine lymph which is at present in use.

He mentions that he has not seen nor heard of a single case of small-pox occurring in any individual who had been vaccinated at the Breslaw Royal Vaccine Institution.

The excellency of the virus used, and the extreme attention with which the operation has been invariably performed there, can be the only reasons for such immunity. Some years ago, vaccination used to be performed in different parts of Germany not unfrequently by clergymen and midwives. Surely, then, it cannot be wondered at, that so many apparently unsuccessful cases have occurred during the late epidemics of variola. "According to my experience," says Dr. E. "the proportion of those cases, wherein *genuine* variola attacks persons who have been properly vaccinated, is nearly the same as that which was announced to be the case by the early English vaccinators at the close of last century, being about 1 to 500; while the proportion of the cases, in which a second attack of small-pox has taken place is about 1 in 800."

Whenever there is any doubt as to the genuineness of the pock induced by vaccination, the surgeon should invariably repeat the operation.

In the last annual report of the Berlin Infirmary, published by Dr. Kuhk, we find that ninety-six cases of "pocky disease" were admitted during the course of the preceding year. Eleven of these proved fatal. Twenty-four were examples of genuine variola; 21 of modified variola; and 51 of varicella. Fifteen of the cases of the genuine variola occurred in persons who had never been vaccinated, and nine in vaccinated persons. Among the former number, was one of a second attack of the disease. The eleven fatal cases occurred in persons who had not been vaccinated. The varioloid and varicellar cases were generally very mild, many of them not requiring any medical treatment.

The period of the variolar disease, which was most dangerous, was at the accession of the secondary fever, which was apt to assume a malignant typhoid type.

Mr. Lawrance. Surgeon to the Royal Military Asylum at Chelsea, has communicated the results of the observations made there during the last thirty years, respecting small-pox, and the protection afforded against it by vaccination, The total number of children, who had been admitted into the hospital, up to the middle of 1835, is 6251; out of which number, 2532 had had small-pox previous to their admission; 3080 had been previously vaccinated; 630 were vaccinated at the Asylum; and nine had neither been vaccinated, nor had ever had small-pox. There had been 62 cases in all of small-pox, observed in the asylum: of these, 26 were after reputed small-pox, 24 after reputed vaccination, three after vaccination performed at the asylum, and nine in patients who had not undergone either vaccination or small-pox.

The total number of deaths was *five*; three occurring in patients who were reported to have had small-pox previously; and the other two, in patients who had never been vaccinated, nor had small-pox before. "No fatal case of small-pox after vaccination has ever occurred in this institution, although there have been 27 cases of the disease among those previously vaccinated."

With respect to re-vaccination, Mr. L. observes that "in no instance in his practice has a genuine vaccine vesicle been produced a second time—merely more, or less inflammation, and sometimes a small pustule, similar to what a thorn introduced into the arm might have caused."

Mr. Field, surgeon to Christ's Hospital, in a paper, which was read before the College of Physicians, has adduced the following very interesting results of his experience at the hospital, during the last ten years. "The total number of children admitted into the school from 1825 to 1834, has been 1877; and it is to be observed that very few children are received, who, from not having undergone vaccination, are susceptible of natural small-pox. The total number of children, who have during that period had the small-pox after vaccination has been 89. The number affected has varied much in different years, as the following table will shew.

| | |
|---|---|
| 1825 | 33 |
| 1826 | 4 |
| 1827 | 12 |
| 1828 | 3 |
| 1829 | 7 |
| 1830 | 4 |
| 1831 | 8 |
| 1832 | 2 |
| 1833 | 15 |
| 1834 | 1 |
| | 89 |

One only of these 89 cases proved fatal. The boy was just recovering from a very severe attack of measles, at the time when he was seized with small-pox. It thus appears that out of 1877 children admitted into the hospital during the last ten years, only one has died of small-pox; that disease, which was formerly such a terrific and devastating scourge.

Mr. Taylor, surgeon at Cricklade, informs us that some time ago he vaccinated several persons with lymph derived directly from the cow, with the view of comparing the vesicles thus induced, with those which follow the insertion of the lymph obtained in the ordinary way from the arm of another patient. He states: "The characters of the two sets of vesicles was perfectly alike;" and again "there is no difference observable in the vesicle produced immediately from the cow, and the one produced from lymph obtained from the Vaccine Institution."

Mr. Taylor, as well as some other correspondents of the Gazette, have communicated several cases, to prove the advantage of immediately vaccinating persons, who have been or still are exposed to the contagion of small-pox, and who may already have been infected with the miasm, although no eruption has yet appeared. The vaccine vesicles under such circumstances will generally be fully developed, and the variolous pustules which may subsequently appear, will be scanty in number, and also greatly modified in character.

None of the cases appear to have proved so severe as that of Mr. Ward's child, related by Dr. Gregory, with the exception perhaps of one mentioned by Mr. Taylor, who states in his report: "vaccination went through all its stages perfectly; small-pox, however, made its appearance, and she had a very full burden." Only one fatal case of small-pox after vaccination occurred at Cricklade, although the number so affected had been very great.

In a large majority of cases the disease was exceedingly mild, scarcely requiring any medical treatment. Mr. T. mentions that in consequence of the fatal case, he re-vaccinated a number of families, of which some members, from taking the vaccine disease in its most perfect form, may be considered to have been unprotected and susceptible of variolous infection; while others, and these formed by far the largest proportion of the whole, resisted altogether the effects of the operation. Four ladies in one family alone were found to be unprotected.*

Many parents, who had had all the members of their families regularly vaccinated, having been alarmed by the fatal case, were anxious that their children should be now inoculated with variolous matter. Mr. T. did this in numerous instances; and in all of them a regular small-pox pustule formed at the punctured parts, but without being followed by any constitutional disturbance or general eruption. Mr. T. strongly recommends that vaccination be repeated after a lapse of several years, say from seven to ten.

By an official announcement of the Prussian Government of July last, we learn, that during the year 1834, 44,454 soldiers were re-vaccinated. Of this number, 33,634 exhibited distinct cicatrices of the previous vaccination; 7134 exhibited indistinct, or imperfectly marked cicatrices; and in 3686, no cicatrices were visible.

The progress of the vesicles was regular and complete in 16,679; irregular in 12,287; and in 15,488, the vesicles, or rather the papulæ were quite abortive.

The operation was repeated a second time in 4530 cases; but with effect only in 866 of these.

During the year 1834, there were in all 619 cases of pocky disease, "blattern" (including varicellar, varioloid and variolous eruptions) among the entire body of the Prussian army.

Of these, there were 38 fatal cases of genuine variola: two only of this number occurred in persons who had been re-vaccinated with effect; six in persons who had been re-vaccinated, but in whom the operation failed, and the vesicles were abortive; and the other thirty cases occurred in young recruits, who had not been re-vaccinated at all. The director of the medical department concludes, that he is warranted by these data, to urge on the military surgeons of the Prussian army the propriety of re-vaccinating, even a second or third time, every soldier who may be under their charge, and in whom the operation has not yet been repeated.—*Magazin fur die gesammte heilkunde.*

* Our readers are probably aware that it is very generally believed by medical men, that the ascertained susceptibility of a person to be affected with one of the diseases, variola and vaccinia, implies and indicates the susceptibility to be affected with the other; and *vice versâ*. When, therefore, re-vaccination succeeds, or in other words, when it induces a well-formed vesicle on a person, who had previously been vaccinated at an early period of life, it is presumed that that person was equally liable to be affected with small-pox, if exposed to its miasm. The two viruses appear to be mutually opposed to, and destructive of, each other.

### Disease of the Uterus, benefited by the Use of Hydriodate of Potass—Dr. Clendinning's Observations on this Medicine.

A lady, 46 years of age, married, and mother of one child, had enjoyed good health until within the last three years, at which period the catamenia, which had hitherto been most regular, became suddenly suppressed. The cause of this suppression was a severe mental affliction at the loss of her son, who was drowned in his twentieth year. Her general health became much deranged; she was distressed with dull wandering pains in the region of the kidneys, and in the hypogastrium; her strength was reduced by several attacks of violent menorrhagia—and, during the intervals between these attacks, there was a constant yellow-coloured discharge from the vagina, and considerable pain was experienced during coition.

On examination, the cervix uteri was found to be tender on pressure, and to be considerably indurated. As this patient had, three years before, been afflicted with a syphilitic affection, a gentle course of mercury was recommended. All the symptoms were aggravated by this treatment, and the discharge now assumed quite a sanious character. When Dr. Clarion was first called in to the assistance of this patient, he found her considerably emaciated, and exhibiting the aspect of one labouring under some organic disease. The flesh was loose and flabby; the hue of the surface was of a dull yellow; the features were anxious and wrinkled; the eyes surrounded with a blueish circle; the tongue pale, and, as it were, sodden; the appetite irregular, and often quite deficient, and the bowels constipated. The patient complained of flying uneasiness in the left thigh, and of sharp, darting pains within the vagina. The neck of the uterus being examined by means of a speculum, it was found to be swollen, hard, and irregular, but not very tender when touched; a bloody discharge, of most offensive smell, oozed from the cervix all round. "D'après toutes ces circonstances, on ne pouvait meconnaitre un squirrhe uterin. L'age de la malade, l'ancienneté de son affection, les symptomes graves et caracteristiques qu'elle presentait, me laissaient aucun espoir de guerison, et me determinerent à porter un prognostic facheux."

The treatment consisted in the occasional application of leeches and poultices to the hypogastrium, and in the internal use of demulcents, and of a mild diet. A tepid bath was ordered to be taken every second day, and all exercise to be most cautiously avoided. The leeches were sometimes applied directly on the cervix uteri, by means of the speculum.

The symptoms of irritation having become considerably abated, Dr. C. commenced the use of the ioduretted hydriodate of potash, in the dose of ten drops twice a-day. The dose was gradually increased to fifteen and twenty drops three times a-day, the other parts of the treatment being steadily persevered in as before. The offensiveness of the vaginal discharge was obviated by the chloride of lime wash, and counter-irritation by means of the tartar-emetic ointment, was employed on the hypogastrium and upper parts of the thighs. This treatment was continued for the space of nine months, during which period, leeches were applied directly to the cervix uteri five times, and a bath, "prolongé de plusieurs heures," was used every second or third day. The result was most unexpectedly gratifying —the uterus was reduced to its normal size, and its cervix lost the hardness and irregularity which it formerly had. For the last three years, this lady has enjoyed excellent health. The catamenia have not been seen; "mais les approches de son mari ne determinent plus aucune douleur."—*Journal des Connaissances Médicales.*

*Remarks.* The preceding case is one of practical importance, and may serve to suggest several useful hints in the management of some uterine diseases.

M. Clarion deserves much more credit for the judicious treatment of his patient, than for the diagnosis, and consequent prognosis, which he pronounced at first. The case was most assuredly not one of genuine scirrhus—that disease, we mean, which is characterized by having a tendency to pass into the state of cancerous ulceration. We cannot, therefore, agree with M. C. either in the gloomy prediction which he made—" qu'elle ne laissait aucun espoir de guerison;" or in the reflection which he deduced from the fortunate issue of the case—" qu'elle prouve qu'avec de la perseverance, on peut se rendre maitre du squirrhe de l'uterus deja ancien, sans avoir recours, ni à la cauterization qui si souvent echoue, ni à l'extirpation, qui joint aux inconveniens moraux de toute espèce des operations sanglantes l'inconvenient non moins grand d'offrir peu de surete." We do not mean to affirm that the disease, in this patient, might not have assumed some of the characters of malignancy, had it been neglected or maltreated—we think that it probably might; but that this most unfavourable event had taken place when Dr. C. was first called in, appears to us more than questionable. Uterine disease is, it is well known, of very frequent occurrence, at that period of life when the catamenial discharge is about to cease; and, from the obscurity of the symptoms which so often attends its commencement and progress—from the distressing effects which it induces, alike on the corporeal and on the mental system of the sufferer, and from the extreme danger of some cases, we cannot too strongly impress on the attention of our readers, the exceeding importance of their making themselves acquainted with every point of its history. Accuracy of diagnosis may almost always be attained by a skilful examination per vaginam, and success in the treatment may very often be assured, by following the principles which directed Dr. C.'s practice in the preceding case. Absolute rest in the horizontal posture, the regular repetition of local bleedings, by means of leeches to the groins, over the exit of the round ligament (we do not approve of applying them directly to the cervix uteri)—the employment of warm soothing fomentations and poultices—the regulation of the bowels by mild aperients, especially the oleum ricini—the increasing of the urinary discharge by cooling diuretics, such as nitre, and the spiritus ætheris nitrici, the occasional use of an anodyne at bed-time, and a mild, unirritating diet, will be found to suffice for the cure of very numerous cases.

In such as are more advanced and obstinate, it is useful to try the effects of local irritation on the hypogastrium, or upper parts of the thighs, by means of vesications or epispastics. The tartar-emetic ointment, used of such strength as to produce decided ulceration of the skin, is certainly preferable to ordinary blisters. We have found the application of the strongest aromatic vinegar, to which some extractum belladonnæ has been added, a very useful and easily-manageable substitute in some cases.

Conjoined with the use of these external means, the hydriodate of potass, and some of the other preparations of iodine, have been often unquestionably beneficial. In our practice, we have usually given it along with the decoction, or the syrup of sarsaparilla; and the agreeable results, which we have repeatedly witnessed from this treatment, warrant us in recommending it earnestly to our medical brethren. The modus operandi of this very active drug is certainly not well understood, but that it exerts some peculiar effect on the capillary and absorbent vessels, cannot be disputed. Its effects are decidedly often analogous with those of mercury in many cases of disease—witness its efficacy in the secondary symptoms of syphilis, in dropsies, enlargements of the viscera, &c. and to these may be added chronic inflammation of some textures, such as of the periosteum, articular apparatus, the skin, serous membranes, &c. Dr. Clendinning, the intelligent physician of the St. Marylebone Infirmary (following the suggestions of Dr. Williams, of St. Thomas's Hospital, who read a paper last Summer on this subject, before the College of Physicians), has recently communicated the results of his experience with the hydriodate in periostitis, and other chronic inflammatory affections of the hard membranous tissues. From his papers in the Medical Gazette, we shall make a few extracts.

The first case was that of a young woman, who "constantly complained of dull pain, usually either in the back or front of the head; it was of *some years' standing*, and sometimes alternated for a time with a pain in the left hypochondrium, with which it also sometimes coincided. This pain I treated at different times by cupping, blistering, setons, purgatives, and by steel, bitters, aromatics, ammonia, and other stimulants, and by anodynes and narcotics of various kinds—also by mercury to ptyalism;" but all to no effect. This patient was then placed under the care of Dr. Elliotson, who, "after having failed with many other remedies, succeeded in relieving her by large doses of the hydriodate of potass. Dr. E. attributed the headache to a periostitic affection of the left temporal bone, and the cure, to the remedial power of iodine in periostitis."

Several cases of nodes on the tibia, and other bones, are adduced by Dr. C. to prove the very salutary effects of the hydriodate in dissipating such chronic inflammations. One of these is so interesting, that we shall transcribe it in an abridged form. A middle-aged gentleman was suddenly seized with headache, vertigo, double vision, and other signs of cerebral disturbance. These symptoms, although new to the patient, were believed to be owing to sympathetic cerebral irritation, arising from periostitis of long standing on the vertex and right side, in front of the ear, causing tenderness of the scalp over the whole of the right half, or rather more, of the head. He had for several years been subject to periostitis of various bones, viz. of the sternum, clavicle, acromion, and cranium. Antiphlogistic remedies had at first yielded considerable relief; but of late they had ceased to be of much use. The hydriodate of potass was now exhibited in small doses, to be cautiously augmented. The dose was rapidly raised to ten, fifteen, twenty, and at one time for a few days, to thirty grains thrice a-day. After about a fortnight's use of the remedy, he was free from headache, visual defects, and every other important inconvenience.

Dr. Clendinning having found that the hydriodate was so remarkably beneficial in cases of periostitis, as had been first distinctly proved by Dr. Williams of St. Thomas's Hospital, he was led to extend its use to a disease which is somewhat analogous to it, viz. chronic articular rheumatism. The results of his trials have been very gratifying, as appear from the following reports.

J. D. æt. 30, married, but never affected with venereal disease, has been subject to rheumatism for fifteen years, and, for the last eighteen months, has been unable to work, or to walk without a stick. He has been in St. George's Hospital, and has taken much medicine without advantage. The only part painful at his admission was the right knee, which for twelve months has caused him so much distress, that he has latterly used opium at night, to the extent of fifteen grains at a dose, to mitigate his severe sufferings. The hydriodate, in doses of four grains thrice daily, was now substituted for his former medicines. In 48 hours, a decided improvement was perceptible; he slept for several hours the second night of the iodic treatment, without taking any opium. In ten days, the pain of the knee was entirely removed; he, however, continued the use of the medicine for a few days longer, and then left it off gradually. The details of the other cases reported by Dr. C. are equally satisfactory, and appear to us fully to bear him out in the assertion, that the hydriodate is a most powerful remedy "in certain forms of inflammation." Dr. Williams has unquestionably the merit of having first shewn, that the hydriodate is, in its operation, an antiphlogistic, or corrigent of that state of the bloodvessels on which chronic inflammation depends, as well as a discutient, or stimulant of the extreme absorbents. (May not the same remark be truly applied to the modus operandi of that most universal of all remedies, mercury?) Several French authors had, indeed, previously recommended it strongly in some of the secondary forms of syphilitic disease; but they had used it, rather on empirical than on any *rational* therapeutic grounds. If subsequent experience confirms the recommendations of Drs. Williams and Clendinning, they will have deserved well of practical medicine.

Dr. C. points out some of the advantages of the hydriodate, as an antiphlogistic medicine, over other remedies of the same class. Its use, says he, involves no restrictions of diet or regimen; it occasions no susceptibility of catching cold, like warm-baths, diaphoretics, and mercury; it does not necessarily debilitate; and it is compatible, chemically and medicinally, with almost every sort of substance it may be desirable to combine it with, as with bitters, astringents, opiates, antacids, aperients, and diffusible stimulants; and Dr. A. Thomson and others have proved its medicinal compatibility with chalybeates and mercurials. The inconveniences occasionally attending its use, are either heart-burn, flatulence, nausea, and diarrhœa; or pains in the head and chest. In one case treated by Dr. C. the patient, a young woman, took about 100 grains a day, for several weeks together, without suffering any inconvenience. In a few instances it has induced severe ptyalism, and affected the gums, teeth, and breath, in the same manner as mercury is so apt to do. In this property, says Dr. C., iodine seems to agree with digitalis and some other active substances, which on rare occasions have been known to produce like effects.

It is by studying the physiological effects of iodine and of its different preparations, and by tracing their analogies with the effects of other medicines, that we may hope to improve our knowledge of its modus operandi, and of extending its employment as a therapeutic agent.*

From the facts which we have brought together, it will be acknowledged that iodine is possessed of antiphlogistic, or, to use the language of the Italian school, of contra-stimulant properties. Whether it is applicable to cases of active inflammation has not yet, as far as we know, been ascertained; but we should be inclined to predict that it will be useful chiefly in chronic diseases, in which the inflammation is of a sub-acute and lingering character, and is accompanied either with deposition of solid water, or with effusion of fluid. Its powerfully diuretic properties are well known. A very interesting case of old, obstinate, and confirmed dropsy, which had resisted a variety of treatments, and which ultimately was cured by means of the hydriodate in large doses, was lately communicated by Dr. Bain of Poplar, to the Lancet.

Professor Bouillaud recommends the hydriodate in chronic diseases of the heart.—*Rev.*

---

### M. VELPEAU ON THE DISEASES OF THE LYMPHATIC SYSTEM—STATE OF THE LYMPHATICS IN PUERPERAL FEVER.

Within the last fifty years, no department of pathology has been more satisfactorily studied than that which treats of the diseases of the vascular system. This system, it is well known, includes the two great sections of the sanguiferous and of the lymphatic or absorbent vessels.

The first of these sections is subdivided, according as the vessels are arterial, and excentric, *i. e.* diverging from one central point; or as they are venous and concentric, *i. e.* converging from the extremities to a common centre. The structure or tissue of these two sets of vessels is very widely different; and this difference in structure leads us therefore à priori to anticipate a considerable difference in their pathological changes. Experience justifies the

---

* Our readers will find a few remarks on the analogies which may be traced in the therapeutic effects of mercury, iodine, and arsenic, in the treatment of cutaneous diseases, in the review of Rayer's treatise in our last number. It seems to us, that they all act on one principle, that of stimulating the extreme vessels.

correctness of this conjecture. The veins, we find, are much more frequently diseased than the arteries, in consequence partly of the greater vascularity of their walls, and partly also of their contents being more often deranged and abnormal (denaturé) than those of the arteries. It is to be remembered that the fluid which permeates the veins has already undergone very important changes, since it was discharged purified from the left side of the heart, and that during its return to the centre, it receives various recrementitial matters, the result of an absorption which is probably going on at all times, and at every point of the body. The veins being the channels by which part of the decayed and useless molecules of the body are conveyed into the torrent of the circulation, before being eliminated from the system, it is quite reasonable to expect that they may frequently become morbidly affected.

The attention of pathologists has of late years been most advantageously directed to this very curious subject of investigation, and much interesting information has already been accumulated. But it is not with phlebitis and other diseases of the veins that we are at present to be engaged, but with the morbid conditions of another set of vessels, allied indeed as to function in several respects with the veins, but essentially distinct from them. There is, says M. Velpeau, another organic system which appears to share with the veins the troublesome prerogative of being the conductors of numerous causes or germs of disease to every part of the body, and of assisting in the development of a multitude of lesions, differing in character and in situation, as well as of being the seat of many morbid alterations affecting immediately their own structure. We shall now attempt to illustrate some of the most important of these alterations. As a matter of course, we commence with inflammation; this being invariably the basis or concomitant of all the others.

Like every part of the animal frame, the lymphatic tissue is liable to inflammation of various sorts, as well as of different degrees. It may be either primary and idiopathic, or secondary and symptomatic. It may be induced as the result of wounds and contusions of the lymphatics themselves, or of the contiguous structures. Perhaps, however, the most frequent cause is the accidental introduction by absorption of foreign or abnormal matters, generated during the morbid changes of these structures, into the canals of the lymphatic vessels. If this etiological view be correct, it points out an intimate analogy which may be traced between the pathology of the venous, and that of the lymphatic system. The countless number of the lymphatics in every part of the body must expose them in an especial degree to the operation of noxious agents. Mascagni, and his distinguished followers, MM. Fohmann, Lauth, and Panizza, are of opinion that almost the whole of the cellular tissue and of its "composés" is made up of lymphatic vessels; but, without adopting this opinion to its full extent, we are quite warranted in asserting that every diseased part of the body is necessarily surrounded and traversed by innumerable lymphatics, and that wherever there exists a lesion of structure, there must these vessels be exposed to becoming charged with heterogeneous matters, the tendency of which is to excite inflammation in their walls.—Viewed in this respect, the inflammation of the lymphatics presents two very distinct varieties, according as the diseased focus, "le foyer morbide," and its contents are, or are not, exposed to the contact of the atmospheric air.—When there is no wound, ulcer, or other solution of continuity, by which the admission of the air is permitted, the molecules of the diseased secretion do not in general acquire properties so decidedly poisonous, as when the deposition takes place in open wounds or cavities. The phenomena of psoas abscess, and of wounded or diseased joints, afford an apt illustration of this remark. By some indeed, the rapid aggravation of the symptoms in such cases, when the cavities burst outwardly, has been attributed to the inner surface of the diseased sac becoming affected with inflammation; but certainly this, if a concomitant, is not the sole cause of the mischief. The absorption of the offensive pus by the surrounding veins and lymphatics appears to be immediately excited; and thus the whole system becomes speedily poisoned with the noxious fluid which has been conveyed by them into the circulation. How often do we observe that deep-seated abscesses, and other morbid changes, may continue for a great length of time, without inducing any alarming or even any very apparent symptoms, provided the abscess or lesion be altogether secluded from the contact of the air. Take, for example, some organic lesions of the nervous centres, and of the parenchymatous viscera of the abdomen; and contrast these with cases of the same sort of disease, when it occurs in the lungs, windpipe, intestines, or other viscera communicating with the air. In the former cases, the symptoms very generally are indicative only of disturbed function of the affected organ; whereas, in the latter, the adjacent tissues, the cellular substance, the lymphatic glands, &c. are always, more or less, involved in disease at the same time, and the general system labours with violent irritation. Whether an absorption of the diseased particles goes on at all in the first set of cases, or whether only these particles are less irritating and poisonous, and therefore less apt to induce inflammation of the lymphatic vessels and glands, than in the second set of cases, we are not quite prepared to decide; but certain it is, that the exposure, or seclusion of any morbid deposit from the contact of the external air has a mighty influence in accelerating or retarding the progress of the mischief.

Although the absorption of acrid matters is, we believe, by far the most common cause of inflammation of the lymphatic system, it is not the only one. It may be induced also either from continuity of tissue of other parts already inflamed, or from an obstruction to the passage of the lymph along the absorbent vessels. It is not easy to account for that peculiarity of some punctured wounds, which disposes them, in an especial degree, to be followed by inflammation of the lymphatics. The observation, however, is one of daily experience.

In cases of poisoned wounds, the accident is no doubt attributable to the direct absorption of the virus.

The symptoms which indicate inflammation of the lymphatics are either constitutional or local. The local symptoms vary considerably in their characters, according as the inflamed vessels are merely subcutaneous, or are more deeply seated. In the former case, the evil will be almost invariably found to be caused, or at least preceded by a solution of continuity, either from a wound, or some ulceration, of the integuments; and it will be very generally observed that the appearances of the wound or ulcer have undergone certain changes, before any symptom of inflammation of the lymphatics has occurred. If suppuration has existed, the quantity as well as the qualities of the discharge will be found altered: the lips and circumference of the sore will be affected with an erythematous blush, and the part will be tense and painful. On a careful examination, hardened striæ or cords, extending from the point of the injury in the direction of the lymphatics to the nearest cluster of lymphatic glands will be felt, and in many cases are apparent to the eye. These striæ are generally either of a bright red, or of a purplish hue: they are tortuous, irregular, and more or less interlaced with each other. It is not always in the immediate vicinity of the wound or ulcer, that these striæ are first observed. M. Velpeau has in numerous instances detected them at some distance from the point of injury, before they were discoverable nearer to it. Patches of erysipelatous redness, of greater or less extent, are frequently observed in the lines of the inflamed vessels. These patches gradually extend, and may at length coalesce, so as to form one uniform surface, affecting the whole of an extremity. A sharp, pungent or burning pain is usually felt in the inflamed surface: the swelling at first is often inconsiderable, and the intervals between the inflamed striæ may retain their natural softness and pliancy: but the reverse of this is more commonly observed, and the tumefaction of the subcutaneous tissue speedily follows the first appearance of an inflammation of the absorbents. The tumefied integuments however seldom acquire that extreme tension and hardness, so characteristic of the diffuse inflammation of the cellular substance, which is produced by other causes: the parts, when pressed by the finger, feel rather spongy and œdematous. In short, none of the local symptoms of inflamed lymphatics exhibit usually much intensity and severity; the redness, heat, swelling and pain are by no means commensurate with the gravity of the constitutional symptoms.

Among the characters of inflammation of the superficial lymphatics, we must not omit to mention the swelling, not unfrequently terminating in suppuration, of those glands with which the vessels are connected.

When the deep-seated lymphatics of a part are first inflamed, as is not unfrequently the case after deep punctured wounds, severe contusions, central suppurations, the symptoms are somewhat different from those enumerated above. The pain, deep, pungent and constrictive, is the symptom which first fixes attention: confined to a limited spot for a day or so, it gradually extends in the direction of the chief lymphatics of the part. It is not, says M. Velpeau, a pain which is radiating, linear, diffuse, or paroxysmal: it is fixed, although emanating from different foci, and of unequal severity in different points. The tumefaction speedily follows the attack of pain, and the one symptom seems to partake in some degree of the characters of the other; for the affected part is not swollen uniformly and alike, but rather in distinct masses, or lumps varying in extent and degree. It is easy to recognize that the swelling commences in the deeper parts first; for the skin generally retains its suppleness for some time. As the disease continues, it becomes more tense, and shining, as if it were attenuated and rarified, and it exhibits a milky-white or pale rose colour. The infiltration of the affected part is always more considerable in this, than in the former species of the disease.

Although we have attempted in the preceding remarks to point out the distinctive characters of the two forms of inflammation of the lymphatics, it is to be observed that neither of them usually exists long without being complicated with the symptoms of the other. In the first form, the infiltration and swelling gradually pervade the entire thickness of the affected part; and in the second, the superficial lymphatics become in a similar manner inflamed, as appears from the red striæ, and erythematous patches visible on the skin.

The constitutional symptoms which attend inflammation of the lymphatics, now demand our attention. Very frequently the invasion of this disease is first announced by irregular shiverings, and tremors over the whole body. These shiverings are followed by feverish heat, dryness of the skin, thirst, and the other symptoms of pyrexia. During the continuance of the disease, the systemic disturbance very often assumes all the characters of typhoid or ataxic fever, in consequence probably of the early absorption of "fluides denaturés" into the general circulation. We have not, however, yet made sufficient progress in the humoral pathology of modern times to determine the amount of influence attributable to this cause.

Inflammation of the absorbents is always to be considered as a disease of serious import. When the constitutional excitement runs high, or when great depression and tendency to low delirium attend the invasion of the disease, it may prove fatal, before the local mischief is very serious or extensive.

But more generally the period of danger is that, when the inflammation has terminated in the deposition of purulent matter. In some cases, the pus is diffused and infiltrated through the affected parts; in others, it is more circumscribed, and is contained within cysts more or less complete, and extensive. Unfortunately the former is most frequently the case, the pus following the line of the vessels, and becoming diffused between the different strata of the muscles. The distinct or insulated abscesses usually occur in the sites of the lymphatic glands; but they seldom present the circumscribed hardness which attends phlegmonous collections of matter.

Several of these may be formed at different points of the limb, and then they may successively break and discharge their contents. M. Velpeau has seen as many as fifteen thus gradually develop themselves, one after the other. In some fortunate cases, they speedily heal, after breaking, even although the pus discharged has been unhealthy and offensive; but more frequently, the patient's strength is gradually exhausted, in part from the profuseness of the discharge, from the formation of abscesses in some of the viscera, and partly also from the alteration induced in the circulating fluids from the absorption of the purulent matter. The shiverings, the decomposition of the features, the ague-like paroxysms of fever, the diarrhœa, irregular sweatings, &c. these symptoms indicate that the system is poisoned by the introduction of a septic principle.

In some rare cases, inflammation of the lymphatics appears to terminate in mere induration and thickening, giving rise to an elephantoid enlargement of the affected parts. This termination belongs exclusively to the chronic form of the disease. The lymph of the vessels, becoming infiltrated into the meshes of the cellular substance, gradually acquires a firmer and firmer consistence, so that in course of time, it looks, when examined on dissection, like a lardaceous deposit.

The observations of M. Velpeau, on the treatment of inflammation of the lymphatics need not detain us, as they communicate nothing very novel or important.

He has alluded to the occasional efficacy of mercurial ointment smeared thickly over the affected parts, but he has not spoken at all of a far more potent remedy, we mean the local application of the nitrate of silver, either in substance, or what is preferable, in solution, over the whole extent of the inflamed surface. This agent exerts an unquestionable influence on the deep-seated subcutaneous tissues, as well as on the skin itself; and we believe that many cases of diffuse inflammation of the cellular substance, whether complicated with disease of the superficial veins and lymphatics, or not, may be most advantageously treated with the nitrate, not omitting at the same time appropriate constitutional treatment. The diffused hardness, which generally remains for a considerable time, after the violence of the disease has subsided, is dissipated most conveniently by frictions with an hydriodated ointment.

In the Number of the Archives Generales for March last, M. Duplay has communicated some instructive cases of metro-peritonitis (puerperal fever), in which on dissection, the lymphatic vessels of the uterus, and of the adjacent parts were found filled with purulent matter. In all the cases, there was a sero-purulent effusion in the abdominal cavity, and in most of them, the substance of the uterus had undergone a considerable ramollissement, and purulent infiltration. In more than one half of the cases, pus was found in the branches of the uterine veins; and in two the thoracic duct itself was observed to contain a puriform serosity. The following description of the appearances which the diseased uterine lymphatics exhibited, is interesting to the pathologist. On the interior surface of the womb, as well as immediately underneath its outer peritoneal covering, we observed numerous grey-coloured lines alternately contracted and dilated, extending in various directions, and communicating apparently with each other. The size of these vessels varied from that of a needle to that of a crow-quill.

Here and there they were dilated into cavities or cysts, large enough to hold a pea. These cavities might be supposed to be small abscesses; but an attentive examination of several cases has satisfied M. Duplay, that they are in truth dilated portions of lymphatics, the walls of these being found to be strictly continuous with the walls of the sacs. The diseased lymphatics were usually most readily discovered on the sides of the uterus, where the broad ligaments are attached, and also around its cervix. Sometimes they might be traced along the trunks of the uterine and ovarian veins, anastomosing with other lymphatics proceeding from the ovaries and fallopian tubes, until they reached the lumbar glands. These glands exhibited various morbid appearances—sometimes they were merely swollen, and redder than natural; at other times they were much softened, and infiltrated with purulent deposit. We have already mentioned that pus was found occasionally, even in the thoracic duct; but this is of very rare occurrence. M. Velpeau has recorded one such case, and M. Tonnellé states that he has met with two. The internal surface of the lymphatics, which contained pus, did not exhibit any pseudo-membranes, or any other traces of diseased change in M. Duplay's dissections.

In some cases, it is less difficult than may be imagined by most to ascertain the state of the uterine lymphatics, as they become greatly enlarged during pregnancy, so as even to equal the size of many of the veins. We have already alluded to the morbid changes of the structure of the uterus itself, which were usually found whenever its lymphatics contained purulent matter. The ovaries, too, and the fallopian tubes, were frequently completely softened in texture, and converted into a "putrilage," which broke down between the fingers.

M. D. has never found abscesses in the joints, nor in the liver, lungs, and other distant viscera, when the uterine lymphatics only, and not the veins at the same time, were diseased.—*Archives Générales.*

These remarks of M. Duplay are all confirmatory of the doctrine which has, of late years, been clearly established, that puerperal fever is, in truth, an inflammatory affection of the uterus and its appendages, and more especially of the uterine veins and lymphatics.

There is evidently, in this disease, a strong tendency to the formation of pus within these vessels; sometimes one set, sometimes the other, is chiefly involved in the morbid process. In both cases, the purulent matter is conveyed into the mass of blood, which thus becomes tainted throughout. Unhappily, the local symptoms are often so obscure as to pass unnoticed, while the constitutional energies rapidly sink, from the operation, apparently, of a malignant typhoid fever.—*Rev.**

---

### On the Use of Local Applications to the Pustules of Small-pox.

Very opposite opinions have been held as to the propriety of using any topical applications in the treatment of small-pox, with the view of modifying the course of the symptoms, and of preventing, or at least of diminishing, the disfigurement of the surface. Some physicians have recommended that the face, and other parts which are most sensitive, and, therefore, are most liable to suffer from the cutaneous irritation, should be bathed frequently with a mild anodyne fomentation, such as equal parts of linseed-tea and the decoction of poppy-heads—others have preferred oily applications; and if these be used, perhaps none is altogether so serviceable as the linimentum aquæ calcis, composed of lime-water and linseed-oil; while a third set praise highly the practice of dusting the skin with calamine, or tutty-powder, more especially in that stage of the eruption when the pustules are breaking, and their contents are oozing out.

M. Bretonneau was the first, we believe, who proposed the topical employment of the nitrate of silver, as the most effectual means to arrest the progress of the suppuration, and to prevent the cutaneous pits and ugly scars, which are so frequently left behind after confluent small-pox. He advised that each pustule on the face should be pricked with a gold or silver needle, whose point had been wetted with a solution of the nitrate. A still more effectual method was to remove the apices of the pustules, and then to apply directly a sharp-pointed stick of the solid caustic. M. Velpeau has reported very favourably of the effects of this practice, which he

* We cannot concur with our Reviewer entirely in some of his commendations of the Hydriod. of Potass.—Eds.

adopted in a large number of cases; and M. Guersent, the talented physician of the Hôpital des Enfans at Paris, states that he has frequently succeeded in arresting completely the progress of detached and isolated pustules, and in thus preventing the formation of scars on the face and other exposed parts.

Under the name of the "methode ectrotique,"* M. Serres has recommended the use of the nitrate on a much more extended principle than any of his predecessors. He recommends it, not only with the view of preventing the deformity of cicatrices, but also as a most efficacious means of counteracting the inflammation of the membranes of the brain, of the eye, ear, &c. which so frequently complicates the progress of small-pox. The method, he employs, varies, according as he wishes to act on isolated pustules, or on a mass of confluent ones. In the former case, he uses a solid stick of the caustic, which he applies to each pustule (without, however, opening it, as M. Bretonneau has practised); and, in the latter, a solution of the salt, of the strength of from 15 to 45 grains to the ounce of water. If the lotion excite much tingling, the part may be bathed with tepid water, and afterwards smeared with olive oil. Ten or twelve hours after the cauterization, it may be necessary to apply leeches to the neck repeatedly, if there is much swelling of the face. When the cauterization of a mass of pustules is superficial, it opposes the development of the pustules, but it does not arrest the progress of the disease. There exists always, under the black crust which is formed, a serous oozing; and when the crust separates, we frequently observe the traces of pustules which have passed through their various stages. When the solution applied is much stronger, it acts as a caustic to a considerable depth; the epidermis and pustules are desiccated, and the case becomes one of a simple burn, "au second degré," attended with very considerable pain; but the constitutional disturbance, nevertheless, may be equally severe, and the patient may sink just as if no local means had been used, supposing the case to have been one of malignancy from the beginning.

M. Damiron agrees so far with M. Serres, that his practice will prevent, in a great measure, the formation of scars and other deformities, so frequently consequent on confluent small-pox; but he does not regard it as a prophylactic of inflammation of the encephalon, as M. Serres has supposed. He alludes, however, to its great utility in arresting the progress of any pustules which may form on the conjunctiva or cornea, and which, it is well known, often induce great irritation, and even destruction of the eyeball and lids, and ultimately total blindness, in many cases.

In the course of the preceding year, some experiments were made at the Hôpital de la Pitié, to ascertain the "ectrotic" effect of some other applications to variolous pustules. The "emplatre de Vigo, cum mercurio,"* softened with olive-oil, was found to be the most convenient and efficacious. Whenever this had been applied, the pustules were not only less numerous than elsewhere, but many of them "sont avortées," without having proceeded to suppuration, and the subsequent desquamation was observed to be much sooner over. When the pustules had begun to suppurate before the plaster was applied, it was remarked that frequently their contents were absorbed more or less completely, and that the subsequent course of the malady was altogether more favourable than is usual under the common treatment.

The simple mercurial ointment, and

* This word is derived from the Greek verb, εκτιτρωσκω, to cause abortion.

* The simple "emplatre de Vigo" is made by boiling lard, wax, turpentine, litharge, oil, and vinegar together, adding some essential oil as it cools; the "e. de Vigo, cum mercurio" is ordered to be prepared by incorporating some quicksilver, previously extinguished in liquid styrax and turpentine, with the simple plaster, softened by heat.

an ointment composed of litharge and lard, were sometimes substituted for the "emplatre de Vigo cum mercurio," and with nearly equal benefit.

If the practice of other physicians confirms the statement of M. Serres, he will have deserved well of medical science. We are assured that the "avortement" of the pustules is never followed by any disagreeable symptoms; and that just in proportion to the extent of the arrest of the local distress, so is the constitutional suffering rendered more mild and favorable.—*Archives Générales, et Dictionnaire de Medecine.*

---

### On the Symptoms and Treatment of Gall-stones.

Case 1. *Grief of Mind—repeated Attacks of Jaundice—Cure by Purgatives.*

Mad. M., 28 years of age, having for the 24 hours previously suffered from severe pains in the epigastrium and back, was seized on the 21st July with violent efforts to vomit. A quantity of yellow bile was rejected, and the patient felt herself easier. But in the evening a shivering came on, the pains returned, the skin became jaundiced, and the whole system was feverish. Veal-tea, and a mixture containing æther and laudanum, were administered. On the 24th, the icteric symptoms had disappeared, apparently in consequence of a profuse perspiration, which had stained her chemise of a yellow colour. The urine was high-coloured, thick, and left an oily deposit on the vessel. On the 5th of August, all her distress returned; the pains were so severe as to cause her to double up the body, and to press with all her might on the epigastrium: the shiverings affected every part of the frame.

*Prescription*—Chicken-tea made into an emulsion: eighteen leeches to the anus. Mixture composed of lettuce-water, lemon-syrup, carbonate of potass, and a few drops of laudanum. Warm bath and repeated emollient and anodyne lavements.

Between this date and the 3d of December her health was never quite established. At intervals the pains, accompanied with vomiting and jaundiced state of the skin, frequently returned. Ætherial potions, combined with laudanum and castor, were used with advantage, and an opiate hemlock plaster was applied on the epigastrium. The catamenia remaining absent, repeated leechings from the vulva were ordered. But these means afforded only temporary relief, and M. Bricheteau determined therefore to try a course of purgatives, "dont j'avais jusque là redouté les effets." No sooner had a senna aperient mixture begun to act briskly, than several small biliary calculi were discovered in the motions: they were of a brown colour, and uneven, and very friable. From this date, the lady's health was speedily improved, and eventually she recovered completely her embonpoint.

Two years after the cure, Mad. A. had a severe attack "de ce mal terrible," which M. Bricheteau quickly removed by the application of ice to the right hypochondriac region. (!)

*Remarks.* The preceding history furnishes a fair specimen of the inefficient and even pernicious effects of French practice in a disease, which no British practitioner would dream of treating in any other way, than in that which effected the cure; we mean by administering active purgatives, either alone, or in combination with antispasmodics.

Case 2. *Mental Distress—Frequent Attacks of Epigastric Pain during several years—Cure by the Application of Ice to the Abdomen.*

Mad. R., 45 years of age, had had repeated attacks of pain in the epigastrium, which was sometimes so severe as to bend her almost double, and force her to scream out. The only relief she obtained during the paroxysm, was from very firm pressure made with both hands on the seat of the suffering. M. B. was called to her assistance on one occasion, and "after," says he, "trying various remedial means ineffectually, I thought of applying one bladder full of ice to the epigastrium, and another to the back. Judge of the patient's surprise, on being relieved from an agony which had lasted 36 hours, by the time that the ice was completely melted." For five years this lady remained free from any similar attack; and on being, after that lapse of time, again seized, M. B. employed the same means with the like magical success.

[No mention is made of any biliary calculi having ever been voided, but the author prognosticates their presence, from the circumstances, that his patient was of an extremely bilious constitution, that she had had usually an attack of jaundice after each attack, and that her disposition was hasty and irascible.—*Rev.*]

Case 3. *Repeated Attacks of Umbilical Pain—Cure by Purgative Lavements.*

Mr. M., 67 years of age, of a bilious constitution, and who had spent an anxious and laborious life as a bookseller, was seized in April, 1828, with acute pain near the navel, which lasted for five hours, and at length was relieved by frictions with oil and laudanum. On examining the abdomen attentively, M. B. discovered that pressure on the epigastrium excited pain, and moreover that there was an unusual resistance to the hand in the situation of the pyloric end of the stomach. This symptom taken in connexion with the frequent vomitings of a black-coloured matter, and other signs of stomach distress, caused M. B. to have fears of the presence of some organic lesion of that organ. Diverse remedies, such as frictions with antimonial ointment on the epigastrium, baths, and some local opiates, dissipated the hardness and resistance of the epigastrium.

In July, 1829, the patient experienced a most severe attack: the pain was felt chiefly in the right hypochondrium, and accompanied with a spasmodic state of the abdominal muscles, so rigid that the plane of the belly was as hard and resisting as a deal board, and so agonizing, that the patient twisted his body into various positions, squeezing the stomach with both hands, and screaming out for relief. These attacks terminated usually by vomiting of a quantity of bilious and mucous matters.

A purgative lavement having been administered, a number of small biliary calculi were voided with great relief to all the symptoms. Baths, soothing and antispasmodic drinks, a milk diet, Seltzer water alone, or "coupé" with beer, and "le remede de Durande" (composed of two parts of the spirit of turpentine and three of sulphuric æther: dose two scruples) were prescribed. As each attack subsided, M. B. always ordered a purgative enema, which almost uniformly had the effect of provoking the discharge of several biliary calculi.

This old gentleman gradually recovered his health, and although still subject to occasional relapses, the attacks were never in future so severe, as they had wont to be.

*Remarks.* Part of the dietetic regimen ordered in the preceding case is reprehensible. Milk and beer are two of the very worst articles of food for a bilious person.

Is it not strange that M. Bricheteau always deferred the use of purgative injections, until the attacks began to abate? Had they been used at an earlier period, and especially along with purgative medicines taken by the mouth, we are quite confident that the patient's sufferings might have been much abridged.—*Rev.*

Case 4. *Vomiting of a Biliary Calculus.*

Madame G. aged 49 years, accustomed to a very sedentary life, and frequently the victim of great mental distress, was seized in June, 1833, with excessive tearing pains in the epigastrium, which was so sensitive, that the lightest pressure was insupportable; and the abdominal muscles were so powerfully retracted, as to leave a deep hollow in the centre of the abdomen, large enough to admit an orange. The sufferings were occasionally so intense, as to deprive the patient of all consciousness, and to force a cold perspiration from every pore: "cette infortunée appellait la mort à grands cris."

The only treatment which had afforded any relief, was the employment of local and general bleedings, and the use of opiates and antispasmodics, in conjunction with warm baths. As the catamenial flow had been very irregular for some time past, the occasional application of leeches to the vulva, aided by the use of revulsive foot-baths, was resorted to. During one of the severe paroxysms of abdominal pain, this lady vomited a firm rounded substance, of the size of a filbert. It was of the consistence of slightly dried clay, was very friable, had a yellowish-grey colour, and afforded on chemical analysis a large quantity of adipocire.

The expulsion of this substance was accompanied with dreadful colic, and followed by a copious alvine evacuation of bilious and mucous discharges, containing a quantity of " diffused solid matter, similar in appearance to what had been rejected by vomiting." The symptoms gradually, but very slowly abated. In a subsequent attack, which this lady experienced, great relief was obtained by applying a blister on the epigastrium, and sprinkling the excoriated surface with a quarter of a grain of acetate of morphia blended with a little lard.

In the course of an hour, the pain was considerably mitigated, and soon ceased entirely. On the morrow it returned, but with less severity. " For five successive days these applications were continued with an always increasing advantage."

*Case* 5. A young lady 26 years of age had during several years suffered frequent returns of most severe epigastric pain, which had been supposed to be of an hysterical character. In one of these attacks, M. B. was called, and he found his patient writhing under excruciating pain in the right hypochondrium, which was so tender that the lightest pressure was insufferable. The attitude which was most easy, was by drawing up the limbs to the belly, and bending the head and body forwards, so as to relax as much as possible the abdominal muscles. She had been in this state of extreme suffering for twelve hours. Various remedies, as æther, the drops of Durande, purgatives, bleeding, &c. had been successively employed, with but little benefit, until the patient voided several small biliary calculi, of the size of large pins' heads: when thrown on a live coal, they burned away with a bright flame, and with a slightly mucous smell. Laxative drinks and enemata encouraged the discharge of more of these calculi, and the patient ultimately was restored to good health.

*On the Symptoms of Biliary Calculi.*

The signs which attend the early formation of these calculi are usually very vague and indecisive. The patient has probably been subject for a length of time previously to bilious attacks, attended with acute pains in the epigastric or right hypochondriac region, extending to the back and shoulders, greatly aggravated by stretching the body, and relieved by doubling it forwards, with the knees drawn up, and by firm compression with the hands on the pained part. At other times, the abdomen is so tender, that the gentlest touch aggravates the sufferings. The stomach is irritable, and frequent and painful vomitings are generally present; the skin acquires a jaundiced hue, and the urine is high-coloured and offensive.

These attacks, after continuing for from a few hours to one, two, or more days, gradually abate, leaving the patient exhausted, and icteric, or perhaps affected with actual hepatitis. In the less severe cases, the relief consequent on the discharge of one or of several calculi is so complete, that the patient is able perhaps on the following day to resume his accustomed occupation.

It deserves notice, that a large number of calculi, even of a very considerable size, may be present in the gall-bladder for a length of time, without giving rise to any violent symptoms. How often do we meet with examples of this occurrence in dissecting the bodies of patients, who, we are certain, had never suffered any of the acute sufferings described above. On the other hand, we have strong grounds of belief that many cases of what are called bilious colic, and of cramp of the stomach, are in truth attributable to the passage of gall-stones.

*Treatment.* Antiphlogistic and sedative remedies are seldom, says M. B. of much benefit in relieving the sufferings excited by the passage of biliary calculi. The "remede de Durande" is of use only occasionally. In some cases it has certainly aggravated the distress, by irritating the stomach, increasing the nausea, and inducing a feverishness of the whole system. It was at first highly lauded as an actual solvent of biliary calculi. This chemical medication originated with Fourcroy, who, finding that æther, oils, and alcalis dissolved the calculi out of the body, recommended them as certain specifics.

With respect to purgatives, M. Bricheteau, whilst he admits their great utility in two or three of the preceding cases, is of opinion that they ought not to be administered in the earlier period of the attacks, on the grounds that they are apt to be rejected by vomiting, and also that they cannot possibly be of service, until the calculi have passed into the duodenum. With not a little inconsistency he however advises the external use of the croton oil rubbed on the epigastrium.

The local application of ice to the seats of the pain he strongly recommends.—*Clin. Med. par Dr. Bricheteau.*

*Remarks.* Our readers need not be told that we deem the therapeutic directions of M. Bricheteau to be very faulty. They are indeed utterly unworthy of a practical physician.

The treatment of the disease which is frequently induced by the passage of calculi from the gall-bladder into the duodenum has a two-fold object in view;—first, to relieve the intense pain and spasmodic suffering; and secondly, to promote the passage and discharge of the offending substances. The use of warm-baths, hot fomentations, and the internal administration of a powerful antispasmodic, are the most judicious means in most cases. Occasionally it is necessary to draw blood; for it is well known that syncope is the most potent of all antispasmodics. Where bleeding is not advisable, a mixture composed of tincture of rhubarb, æther, laudanum, and peppermint water, will be found to be of great use in most cases. At the same time, a strong purgative injection will be found to cause a derivation of action from the upper part of the intestinal tube. The injection may be made either of senna and salts, or of spirit of turpentine in barley-water.

Frictions with a stimulating anodyne embrocation on the abdomen are frequently of considerable advantage.—*Rev.*

---

### CASE OF MOLLITIES OSSIUM, AND FATTY DEGENERATION OF THE MUSCLES.

The patient, a woman 61 years of age, was an inmate of the Hospice de la Salpetrière. Two years previously to her admission she had injured her left thigh, which in consequence became inflamed and much swollen. After five months treatment the swelling, &c. was subdued. A year ago, the pains returned without any evident cause in the weak limb; they then extended upwards on each side to the false ribs; and six months later, both shoulders and the right lower extremity became affected. It was with the hope of obtaining relief, that she was admitted into the Hospice in May, 1834. There was no redness, or inflammatory tumefaction visible at any of the pained parts. The pains were always much aggravated by any movements. In the lower limbs, they generally were felt first in the feet, whence they shot up, "comme un eclair," to the pelvis; at other times they were more dull, and diffused over a larger surface. The pain at the lower part of the thorax was sharp and lancinating—that in the shoulders was less violent, and did not radiate to any extent. The limbs were usually semi-flexed; when, however, the pains were severe, they were forcibly contracted. The important functions of the body were but little disturbed, and the intellect was unimpaired.

The acetate of morphia was given with occasional advantage. M. Recamier prescribed a terebinthinate potion, which seemed to agree with the patient; but the pains always retained the same character, of being much aggravated on the slightest motion: in the chest they were sometimes so acute, as to deprive the poor woman of her consciousness while they lasted.

The lower limbs became at length fixedly bent. An attack of bronchitis was the immediate cause of death, which occurred in January, 1835.

*Dissection.* The sciatic, median, radial, ulnar, and pneumogastric nerves were most attentively examined: they were healthy in every respect, with this exception, that the cellular partitions which separated the different fibrils, even the smallest ones, were "envahies" with fatty deposit. Most of the muscles were extremely pale, and of a yellowish colour. A large portion of the proper muscular fibre had disappeared, and its place was occupied with adipose tissue, which might be traced "entre les fibres les plus tenaces." This fat was everywhere peculiarly dry to the touch, and looked as if it had been long exposed to the air.

The vertebræ, the clavicles, the bones of the arm, thigh, and pelvis were so soft as to yield to the knife. The interior of the bones exhibited, almost every where, "un tissu areolaire trés-rarifié," of a greyish-white colour, containing a quantity of a gelatiniform deposit. In some points, as at the neck of the femur and in the left os ilii, the osseous structure had degenerated into a reddish detritus, occupying the centre. None of the joints presented any very obvious morbid appearances.

*Observations.* During the life of the patient, her disease was supposed to be chronic rheumatism, complicated with some degree of neuralgia. In considering the organic etiology of the case, we ought to remember that the muscular tissue has a tendency, in bed-ridden patients, to undergo a partial transformation into fatty matter. Some pathologists object to the term "transformation," on the ground that the morbid appearance is attributable, not to an actual conversion of the one tissue into the other, but only to the deposition of fatty matter in the room of the muscular fibre, which had previously become atrophied and absorbed. The subject of the preceding case had been long confined to bed, in an almost motionless position. The analysis of the softened bones indicated that they contained an unusual quantity of a gelatinous substance. The immunity from disease of the joints, the very parts where the pains had been most severely felt, suggests a useful reflection to the practical physician. If the question be put, what probably was the cause of the fatty degeneration of the muscles, and of the gelatinous degeneration of the bones, we should answer—"une grande retrogradation vers leurs élémens primitifs de tous les organes devenus inutiles dans l'économie."

It is a curious feature in the pathology of some atrophies, that certain organs are often found to be converted more or less completely into a fatty matter. The peculiarly fatty condition of the liver in phthisis pulmonalis is an apt illustration of this remark.

---

### Hydatid Cyst, opening into the Intestines and Urinary Bladder—Remarks on Hydatids in general.

The patient, a man, 40 years of age, experienced for the first time, in May, 1828, a sense of weight, accompanied occasionally with colicky pains in the hypogastric region. The abdomen generally was rather full, and, in the left iliac fossa, an indolent swelling, of the size of the fist, could be distinctly felt. In April, 1834, he was admitted into the La Charité Hospital, in consequence of pyrexial symptoms, which had existed for the preceding week. The tumor in the left iliac fossa had extended itself into the hypogastrium; it was as large as a full-sized orange, was slightly painful when pressed, and communicated, on percussion, the feeling of an indistinct fluctuation, and of a "frémissement, ou de collision de corps elastiques, mobiles, comme si on frappait sur un corps elastique." On the following day, he voided by stool a very great quantity of torn hydatids, mixed with pus, and other liquid matters. Some of these discharged cysts must have equalled in size a walnut. For several successive days, hydatids were voided with the stools.

The feverish symptoms rapidly abated, and the swelling diminished in volume. For a month, the patient's health seemed to be much better; but after that period, he was seized with an attack similar to the preceding one; the abdomen again became tense and painful, and the constitution speedily sympathised with the local distress: the bowels were obstinately costive, and the discharge of urine was scanty and attended with pain. After some days suffering, a quantity of hydatids was again passed per anum; and forthwith the pyrexia subsided: the tumor was still hard, tender, but not elastic as before. On the 8th of June, he was seized suddenly with an inordinate desire to void urine; hitherto it had been quite clear, but now it was muddy, purulent and mixed with a gaseous fluid. The patient being much alarmed by bubbles of air escaping from the urethra, mentioned the occurrence to his physician, who satisfied himself of the correctness of the account.

Under the use of appropriate means, the state of the urine became much more natural, and there was no longer any escape of air along with it. The patient left the hospital at this time, retaining however a hard and indolent tumor in the left groin.

*Observations.* With the exception of the bones, there is perhaps no part of the body exempt from the formation of the vesicular entozoa, known by the name of hydatids (from their watery contents) or acephalocysts (from the deficiency of any appreciable head). The liver, womb, kidneys and cellular tissue are the parts in which they are most frequently found.

Numerous cases are on record, similar to the preceding one, in which they have been discharged from the abdomen either by an outward opening through the abdominal parietes, or by vomiting, or lastly by stool. M. Devilliers has narrated a most interesting case, wherein a vast quantity of hydatids was discharged through an aperture in the epigastrium: the woman, who was upwards of seventy years of age, ultimately recovered. Plater and Guatani have published similar cases.

Hydatids have been discharged from the urethra, and numbers of them have been found after death floating loose in the bladder.

In such cases, it is most probable that they have descended from the kidneys along the ureters, or that they have been introduced into it from a cyst formed in the pelvis, which has opened by ulceration into the bladder, as is supposed to have taken place in the preceding case. Their occasional development within the cavity of the uterus, giving rise to symptoms simulating those of abortion, is well known to every obstetrical physician. Dr. Freteau has published in the Journal de Médécine, Chirurgie et Pharmacie, a most interesting example of a number of hydatids having been discharged after an operation of paracentesis thoracis for empyema. M. Cloquet exhibited to the Faculty of Medicine in Paris, nearly 150 hydatiform bodies, varying from one to three lines in diameter, lenticular in shape, and nearly transparent, which he had discovered floating within the bursa mucosa, situated between the tendon of the gluteus maximus, and trochanter major; and since that date, he has met with them in an accidental cyst, near the insertion of the triceps brachii, and also in the sheath of the tendon of the palmaris longus.—*Archives Générales et Dict. de Medecine.*

